# Molecular Modeling of the Raman and SERS Response of 6‑Mercaptopurine: Tautomerism, Surface Coverage, and Charge Transfer

**DOI:** 10.1021/acs.jpcc.6c03520

**Published:** 2026-09-27

**Authors:** Paola Cimino, Pellegrino Musto, Marianna Pannico, Federico Coppola, Nadia Rega

**Affiliations:** † Department of Chemical Sciences, 9307 University of Napoli Federico II, Complesso Universitario di Monte S. Angelo, via Cintia 21, I-80126 Napoli, Italy; ‡ 510496 Institute for Polymers, Composites and Biomaterials, National Research Council of Italy, Via Campi Flegrei, 34, 80078 Pozzuoli, NA, Italy; § Scuola Superiore Meridionale, Via Mezzocannone 4, I-80134 Napoli, Italy; ∥ 657489 Istituto Nazionale Di Fisica Nucleare, Sezione di Napoli, Complesso Universitario di Monte S. Angelo ed. 6, via Cintia 21, I-80126 Napoli, Italy

## Abstract

Surface-enhanced Raman spectroscopy (SERS) of 6-mercaptopurine (6-MP) was investigated by combining the experimental Raman measurements with Density Functional Theory calculations to clarify the effects of tautomerism, aggregation, adsorption geometry, and short-range charge transfer. In aqueous solution the thione forms, Thione­(N_1_,N_7_) and Thione­(N_1_,N_9_), are the only thermally accessible species, whereas the thiol forms are significantly less stable. X-ray derived dimeric models provide closer agreement with spontaneous Raman spectrum, showing that intermolecular interactions are essential for describing the solid-state spectrum; for the best Thione­(N_1_,N_9_) dimer, the mean deviation is 1.1 ± 0.6%. For the adsorbed species, calculations on a tetrahedral Au_20_ cluster reveal that the thermodynamically preferred flat configurations do not provide the best match to the experimental SERS spectrum, which is instead more accurately reproduced by sulfur-bound vertex and center motifs. Increasing surface coverage and mixed-tautomer adsorption are further required to recover the experimental relative intensities, with a 3:1 Thione­(N_1_,N_9_)/Thione­(N_1_,N_7_) model giving the best agreement. Distance-dependent analysis further indicates a short-range chemical-enhancement regime below about 4 Å, marked by charge separation, HOMO stabilization, and selective amplification of vibrational modes localized at the molecule–gold adsorption interface. This work introduces a multitautomer modeling strategy that explicitly goes beyond the conventional single-molecule 1:1 adsorption paradigm, demonstrating that only the concurrent treatment of surface coverage, tautomeric coexistence, and short-range charge transfer yields a physically consistent and quantitatively accurate description of the SERS response.

## Graphical abstract

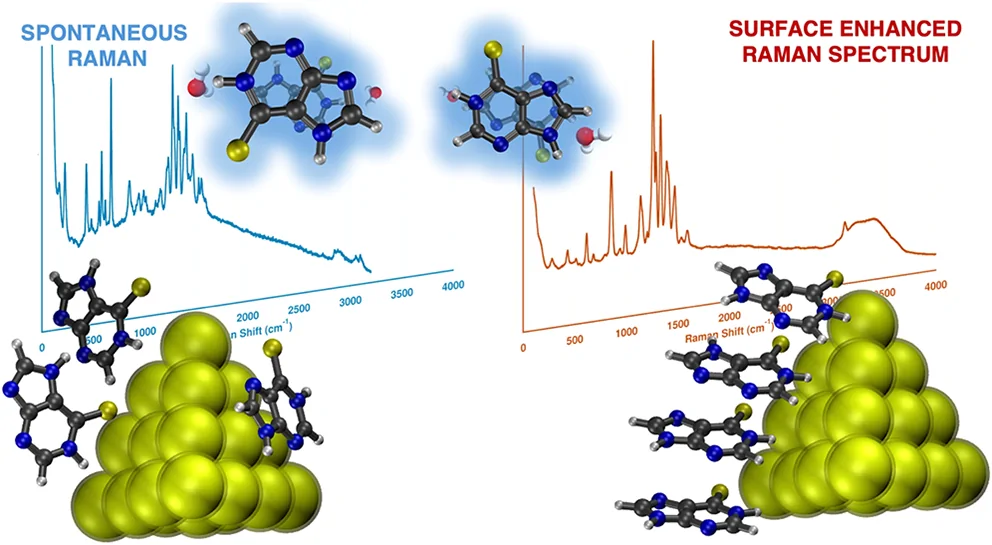

## Introduction

6-Mercaptopurine (6-MP) is a sulfur-substituted analogue of purine and adenine, first synthesized in the early 1950s and approved shortly after as a chemotherapeutic agent for the treatment of childhood and adult leukemias.^1,2^ Today, 6-MP is also employed in therapies for choriocarcinoma, polycythemia, rheumatoid arthritis, and inflammatory bowel disease.^2–4^ Along with methotrexate (MTX), it remains a core component of maintenance therapy for acute lymphoblastic leukemia (ALL), administered daily in pediatric and adult patients.^5–7^

Due to its high cytotoxicity and significant interindividual pharmacokinetic variability—particularly pronounced in pediatric populations—precise dosage control of 6-MP is essential.^6–8^ This is typically achieved through Therapeutic Drug Monitoring (TDM), which involves routine analysis of drug concentrations in biofluids, using techniques such as gas chromatography–mass spectrometry (GC–MS) and high-performance liquid chromatography–tandem mass spectrometry (HPLC–MS/MS).^9–11^

While effective, these methods are costly, infrastructure-intensive, and limited to specialized facilities. Alternative analytical strategies for TDM have thus been explored, including electrochemical,^12,13^ voltammetric,^14^ and optical methods.^15,16^ Among these, Surface-Enhanced Raman Spectroscopy (SERS) is particularly promising for hypersensitive, multiplexed, and minimally invasive analysis.^17–19^

SERS was first observed in 1974 by Fleischmann et al.^20^ and later interpreted by Jeanmaire, Van Duyne,^21^ Albrecht, and Creighton^22^ as arising from electromagnetic and chemical enhancement mechanisms.^23^ When molecules are adsorbed onto coinage metal nanostructures (typically Ag, Au, or Cu), their normally weak Raman signals can be amplified by up to 10^14^ times.^24–28^

Two main mechanisms contribute to this enhancement,^29,30^ the Electromagnetic Mechanism (EM), which dominates in most SERS experiments, and the Chemical Mechanism (CM). The EM mechanism originates from localized surface plasmon resonances (LSPR) that significantly amplify the local electric field in the vicinity of metal nanostructures—particularly within nanoscale junctions or “hot spots”. This field enhancement increases the Raman scattering cross-section by several orders of magnitude. In parallel, the CM mechanism involves charge-transfer (CT) interactions between the metal surface and the adsorbed molecule, which can modify the electronic structure of the system and enable resonance Raman transitions through the formation of metal–molecule hybrid orbitals.^23,31–33^

The landmark discovery of single-molecule SERS in 1997 further demonstrated the technique’s extraordinary sensitivity.^23,31^ Today, SERS represents a versatile analytical tool across a broad spectrum of fields—including, to name just a few, nanotechnology,^34^ catalysis,^35^ biosensing,^36^ and clinical diagnostics, such as intraoperative imaging and liquid biopsy.^32,37^

Density functional theory (DFT) plays a central role in simulating SERS spectra, particularly for understanding the enhancement contributions and rationalizing the experimental signatures.^38–46^ In DFT models, the analyte is typically bound to small metal clusters (e.g., Ag_*n*_, Au_*n*_ or Cu_*n*_), which serve as surrogates for the nanoparticle surface. The key aspects affecting the computed SERS properties include the size and composition of the metal cluster, the adsorption geometry and local site effects, and the quantification of the chemical enhancement.

Larger clusters generally allow for a better representation of collective electronic effects and plasmonic interactions, which translates into significantly higher computed Raman activities and more realistic adsorption geometries.^25^ Moreover, the composition of the cluster plays a critical role: bimetallic systems, such as Ag–Cu or Au–Cu, have been shown to exhibit synergistic enhancements, often attributed to improved charge-transfer efficiency between the mixed-metal substrate and the adsorbate.^47,48^

The adsorption geometry is equally important. The local binding site (e.g., vertex versus face versus edge) and the coordination environment strongly affect the magnitude of the vibrational enhancements, with vertex-bound configurations often showing stronger enhancements.^49^ Furthermore, the adsorption process perturbs the electronic structure of the molecule, modulating its frontier orbitals and thereby selectively enhancing certain vibrational modes, particularly those involved in charge-transfer interactions.

Finally, the quantification of the chemical contribution to the overall SERS signal reveals that static chemical enhancement mechanisms (CHEM) typically result in enhancement factors ranging from 10 to 100, while charge-transfer resonances (CT) can produce significantly higher amplification, with estimated factors in the range of 10^3^ to 10^6^, depending on the specific molecular structure and resonance conditions.^25,50–53^

6-MP is well-suited for SERS studies due to its rich donor environment (four nitrogen atoms and a sulfur atom), facilitating robust adsorption onto noble metal surfaces.^54,55^ Experimental studies have shown rapid adsorption kinetics and strong affinity for gold and silver nanoparticles. Beyond sensing, 6-MP-metal interactions may hold therapeutic relevance in nanomedicine and targeted delivery.^56^

In this work, we combine DFT calculations with experimental Raman and SERS spectroscopy to gain molecular-level insight into the vibrational properties of 6-MP in both solution and adsorbed states. 6-MP is characterized by a rich tautomeric landscape, with at least six chemically plausible prototropic forms. The tautomerization barriers and possible role of solvent-assisted proton transfer are not established yet, making it difficult to define a unique dominant species under the experimental conditions. As a first step, we assess the tautomeric distribution of 6-MP in water by identifying the lowest-energy species: Thione­(N_1_,N_7_) and Thione­(N_1_,N_9_). However, connecting this equilibrium picture to the experimentally observed Raman and SERS spectra is not straightforward.

For this reason, in the present work, we tried to rationalize the main spectral lines and the coupling modes of the experimental Raman spectrum in the fingerprint region and we evaluated different types of models. In order to consider the possible interactions between the same tautomers and between tautomers and some water molecules, we extracted two dimeric units from the X-ray crystal structure—including crystallization water molecules—with, one with Thione­(N_1_,N_7_) and another with Thione­(N_1_,N_9_) of 6-MP. Crystallographic data report different solid-state form (characterized also by the presence, or not, of crystallographic water molecules) in which distinct Thione tautomers are present.^57–59^ This approach provides a valuable framework for disentangling the contributions of distinct tautomers and supramolecular motifs to the observed vibrational response. We then extend our analysis to simulate the SERS spectrum of 6-MP adsorbed on a tetrahedral Au_20_ nanocluster (Figure 1), which serves as a minimal three-dimensional model of the nanoparticle surface, focusing on the spectral changes associated with different binding modes and coverage levels.

**Figure 1:**
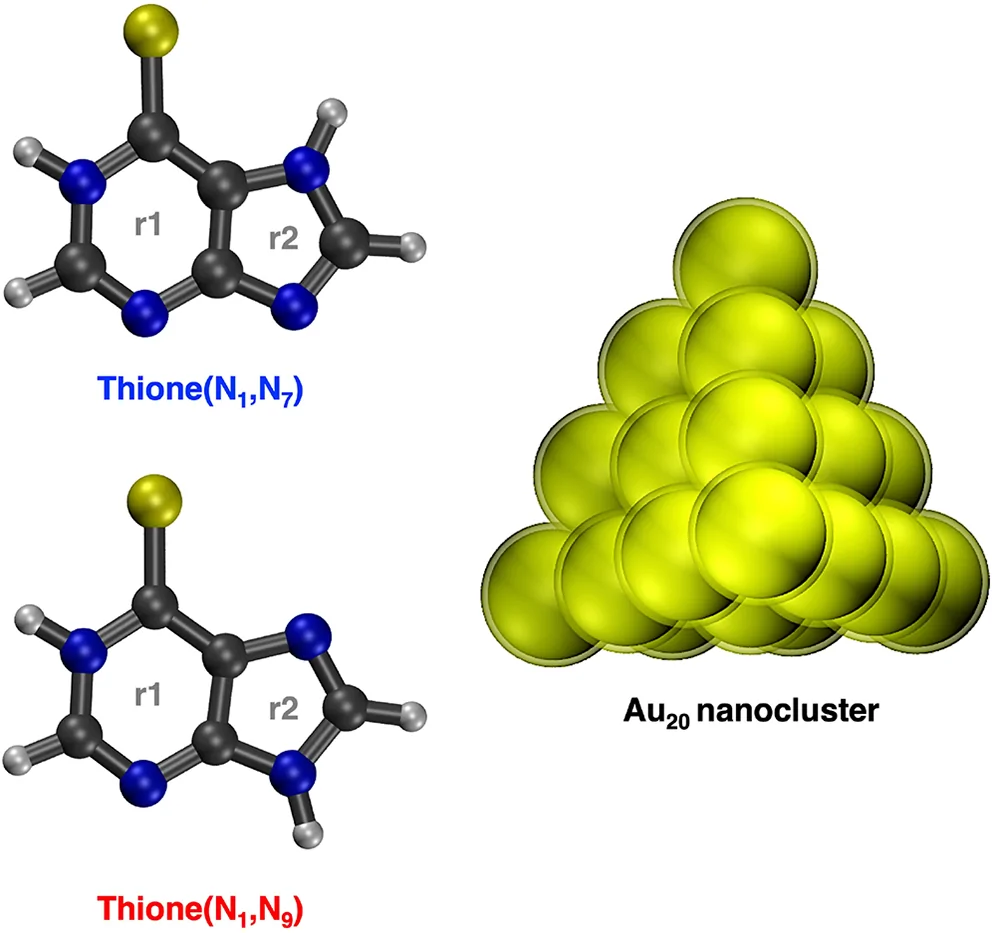
Left: the most stable 6-mercaptopurine tautomers (named Thione­(N_1_,N_7_) and Thione­(N_1_,N_9_)) which differ in the position of the proton attached to the purine ring nitrogen atom. Right: the tetrahedral Au_20_ cluster employed as a representative model of the gold nanoparticle surface in all adsorption studies presented in this work. Ring 1 (r1) and ring 2 (r2) are also labeled.

Our findings reveal that a three-dimensional nanoparticle model is essential to capture the complex enhancement patterns observed experimentally. Moreover, by explicitly analyzing multimolecule coverage, we unveil cooperative effects that modulate spectral intensities beyond the intrinsic polarizability changes.

Taken together, this study provides new insights into the fundamental aspects of 6-MP adsorption, SERS enhancement mechanisms, and the role of noncovalent interactions in shaping vibrational spectra. The combination of theory and experiment sets the stage for future applications of SERS in pharmacological sensing and precision diagnostics. Crucially, this work moves computational SERS modeling beyond the widely adopted single-molecule representation. By explicitly constructing and evaluating heterogeneous multimolecular adsorption models, featuring up to four 6-MP units in different tautomeric ratios anchored to distinct surface sites, we establish a new methodological benchmark for the molecular-level simulation of SERS spectra of flexible, multitautomeric analytes on realistic nanoparticle models.

## Methods

### Experimental Details

#### Materials

Trisodium citrate (TC), gold­(III) chloride trihydrate (HAuCl_4_·3H_2_O), ascorbic acid (AA), sodium borohydride (NaBH_4_), Cetyltrimethylammonium bromide (CTAB), ethanol, *p*-Mercaptoaniline (pMA) and 6-mercaptopurine (6-MP) were purchased from Sigma-Aldrich. Milli-Q water was used for the preparation of gold nanoparticles.

#### Synthesis and Surface Functionalization of Gold Nanoparticles

The AuNPs were synthesized in aqueous solution by using the seed-mediated growth method as described in our previous work.^60,61^ Once prepared, the AuNPs solution was stirred for 10 min and then centrifuged three times at 13,000 rpm at 35 °C for 20 min. The AuNPs were resuspended in water and kept at room temperature for further characterization. The SERS performance of the synthesized AuNPs was evaluated using the enhancement factor (EF) as a figure of merit. EF was evaluated by comparing the spontaneous and the surface enhanced spectra of pMA according to EF = (*I*_SERS_·*N*_REF_)/(*I*_REF_·*N*_SERS_) where *I*_SERS_ is the integrated area of a specific SERS signal (at 1074 cm^–1^) and *I*_REF_ is the integrated area of the corresponding Raman signal (at 1088 cm^–1^), both normalized for exposure time. Analogously, *N*_SERS_ and *N*_REF_ represent the number of molecules contributing to the SERS and the Raman signal, respectively, and were evaluated according to the procedure described in refs 60,61 . The AuNPs colloidal solution was functionalized at room temperature with either a 100 μM pMA/ethanol solution or a 20 μM 6-MP/water solution, added in a 1:1 volume ratio under continuous magnetic stirring (10 min).

#### Raman Spectroscopy

The Raman spectra were collected by an XploRA Plus confocal Raman microspectrometer (Horiba-Jobin Yvon, Edison, NJ, USA) operating with a 638 nm diode laser as exciting source. The 180° backscattered radiation was collected by an Olympus metallurgical objective (MPlan 10×, NA = 0.28, laser power 25.4 mW) with a 1200 grooves/mm grating and confocal and slit apertures set to 500 and 200 μm, respectively. The radiation was focused onto a CCD detector (Syncerity) cooled at −60 °C by a Peltier module. The reference spectrum of pMA was collected on a 10 wt % pMA ethanol solution by using a quartz cuvette with chamber volume of 700 μL (Hellma GmbH & Co, Jena, Germany) and 10 s of acquisition time. The pMA SERS spectrum in the colloidal solution was collected in the same operating conditions. The spontaneous 6-MP spectrum was collected by dropping a 100 μM solution of 6-MP in water, after complete drying. The spectrum was acquired by an Olympus metallurgical objective (MPlan 50×, NA = 0.50) with 2 s of acquisition time, averaging 10 consecutive scans. The SERS spectrum of 6-MP was collected on a 20 μM solution in the same operating conditions of the pMA SERS spectrum but with a 1 s of exposure time and 10 accumulations.

#### Absorption Spectroscopy

The UV-vis spectra of 6-MP were recorded on a single-monochromator UV-vis spectrophotometer (V-570, Jasco, Easton, USA) using a 1.00 cm quartz cuvette, with a scan speed of 400 nm/min over the wavelength range of 300–800 nm. For the quantitative analysis of 6-MP, a UV calibration curve was prepared by diluting the 6-MP stock solution to obtain a set of seven standards ranging from 120 μM to 1 μM.

### Computational Details and Methodologies

All conformers of 6-mercaptopurine (6-MP) were fully optimized using Density Functional Theory (DFT) using the Becke, three-parameter, Lee–Yang–Parr hybrid functional (B3LYP)^62–65^ in combination with a Pople-type triple-ζ split-valence basis set including diffuse functions on non-hydrogen atoms and (d,p) polarization functions for heavy and hydrogen atoms, respectively (6–311+G­(d,p)). To account for long-range dispersion interactions, we included Grimme’s D3 empirical dispersion correction with Becke–Johnson damping (GD3BJ),^66^ as implemented in the Gaussian 16 software package (G16.C01).^67^ Harmonic vibrational frequency calculations were carried out to confirm the nature of stationary points as true minima on the ground state potential energy surface (i.e., no imaginary frequencies), and to obtain both infrared (IR) and Raman intensities. To simulate solvent effects, all calculations were carried out in water using the conductor-like polarizable continuum model (C-PCM).^68,69^

The crystallographic structures of 6-mercaptopurine were retrieved from the Cambridge Structural Database (CSD)^70^ in order to account for its known solid-state variability arising from prototropic tautomerism and hydration-dependent packing. In particular, two monoclinic crystal forms were considered: (i) a distinct hemihydrate crystallizing in space group *P*2_1_/*n* (No. 14), characterized by *a* = 9.3608 Å, *b* = 11.0691 Å, *c* = 13.0914 Å, α = 90°, β = 110.300°, and γ = 90° (unit-cell volume *V* = 1272.22 Å^3^, CSD deposition number 1447142)^58^ and (ii) a monohydrate crystallizing in space group *C*2/*c* (No. 15), with lattice parameters *a* = 15.3600 Å, *b* = 7.7461 Å, *c* = 12.3762 Å, α = 90°, β = 101.526°, and γ = 90° (unit-cell volume *V* = 1442.83 Å^3^, CSD deposition number 998661).^57^ Both crystal structures preserve the thione (C = S) form of the molecule but differ in the location of the second ring proton and in the hydrogen-bond topology imposed by the different water contents. For each crystal, representative dimeric motifs (including the relevant crystallization water molecule(s), when present in the local environment) were extracted to construct the initial models. Geometry optimization and harmonic vibrational frequency calculations were then carried out at the B3LYP/6–311+G­(d,p)/GD3BJ/C-PCM level of theory, enabling a detailed interpretation of the experimental Raman spectrum in the solid state. The optical properties of all six tautomeric forms of 6-MP were investigated within the framework of time-dependent density functional theory in the Linear Response regime (LR-TDDFT). To account for solvation effects in a physically meaningful yet computationally affordable manner, a hybrid explicit/implicit solvation model was adopted, in which four water molecules were explicitly included around each tautomer while the bulk solvent was described using the C-PCM continuum model. Vertical excitation energies and oscillator strengths were computed for the lowest 50 singlet excited states for each tautomer, employing the same level of theory used for the ground-state geometry optimizations.

For the study of 6-MP adsorbed on gold cluster (6-MP@Au), we modeled the interaction using a 6-atom (Au_6_) and a 20-atom gold cluster (Au_20_), this latter a commonly adopted model in theoretical SERS studies to mimic nanoscale metallic surfaces. Optimizations and frequency calculations for these hybrid systems were conducted at the same B3LYP/GD3BJ/C-PCM­(water) level of theory, employing a mixed basis set: 6–311+G­(d,p) for 6-MP species, while the LANL2DZ (Los Alamos National Laboratory 2 double-ζ)^71^ effective core potential—ECP—and basis set were used for the gold atoms. Natural Bond Orbital (NBO) analysis was also performed using the NBO7 program to gain insight into the electronic structure and donor–acceptor interactions in each adduct.^72^ For the plots of simulated Raman spectra, pure Gaussian band shapes were used with a FWHM of 10 cm^–1^, unless otherwise stated, and were scaled by a uniform factor of 0.9613 to compensate for the systematic overestimation of vibrational frequencies arising from both the harmonic approximation, which neglects vibrational anharmonicity, and residual inaccuracies in the electronic-structure method and basis set.

The Raman Scattering factors (RSF_*i*_, see eq 1) calculated with the G16.C01 program

1 $$\mathrm{RS}F_{i}=45{\left(\frac{d\mathrm{\alpha ̅}}{dQ_{i}}\right)}^{2}+7{\left(\frac{d\gamma }{dQ_{i}}\right)}^{2}$$

where

- RSF_*i*_: Raman scattering factor for the *i*th normal mode (units: Å^4^/amu);
- α̅: isotropic polarizability, defined as $\mathrm{\alpha ̅}=\frac{1}{3}\left(\alpha_{\mathrm{xx}}+\alpha_{\mathrm{yy}}+\alpha_{\mathrm{zz}}\right)$;
- γ: anisotropy of the polarizability tensor, defined as
$$\gamma^{2}=\frac{1}{2}\left[{\left(\alpha_{\mathrm{xx}}-\alpha_{\mathrm{yy}}\right)}^{2}+{\left(\alpha_{\mathrm{yy}}-\alpha_{\mathrm{zz}}\right)}^{2}+{\left(\alpha_{\mathrm{zz}}-\alpha_{\mathrm{xx}}\right)}^{2}+6\left(\alpha_{\mathrm{xy}}^{2}+\alpha_{\mathrm{yz}}^{2}+\alpha_{\mathrm{zx}}^{2}\right)\right]$$;
- *Q*_*i*_: normal coordinate of the *i*th vibrational mode;
-
$\frac{d\mathrm{\alpha ̅}}{dQ_{i}}$ : derivative of the isotropic polarizability with respect to *Q*_*i*_;
-
$\frac{d\gamma }{dQ_{i}}$ : derivative of the polarizability anisotropy with respect to *Q*_*i*_;

were subsequently converted to relative Raman intensities (RI_*i*_, see eq 2) using the following relationship derived from the intensity theory of Raman scattering^73–76^

2 $$RI_{i}=\underset{f}{\underset{︸}{{\frac{\left(2\pi \right)}{45}}^{4}\cdot \frac{h}{8\pi^{2}c{\mathrm{\nu ̃}}_{i}}}}\cdot {\left({\mathrm{\nu ̃}}_{0}-{\mathrm{\nu ̃}}_{i}\right)}^{4}\cdot \frac{1}{1-e^{\left(-\mathrm{hc}{\mathrm{\nu ̃}}_{i}/k_{B}T\right)}}\cdot \mathrm{RS}F_{i}$$

Here, *h*, *c*, *k*_B_, and *T* are the Planck constant, light speed, Boltzmann constant, and Kelvin temperature, ν̃_0_ is the exciting frequency (in cm^–1^: 638 nm = 15673.981191 cm^–1^), ν̃_*i*_ is the vibrational wavenumber of the *i*th normal mode and, *f* is a suitably chosen common normalization factor for all peak intensities.

## Results and Discussion

### Experimental Spontaneous Raman and SERS Spectra

The Raman spectrum of crystalline 6-MP (Figure 2, left panel) displays a rich pattern of peaks in the fingerprint region, while showing only very weak features in the ν­(CH)/ν­(NH) range. The SERS spectrum displays a similar appearance, with the prominent signals localized in the 800–1600 cm^–1^ interval. Only a weak doublet is detected above 3000 cm^–1^ (Figure 2, right panel). A direct comparison between the two patterns is reported under Figure S1 in SI. The peaks in the Raman spectrum are significantly sharper than those in the SERS spectrum, a consequence of the highly ordered crystalline structure of 6-MP compared to the less organized adsorbate in the colloidal solution. Another notable observation is that the main difference between Raman and SERS spectra lies in the relative intensities of specific bands, especially below 700 cm^–1^, whereas the positions of most peaks remain within a few wavenumbers. This indicates that 6-MP adsorbed on the Au surface retains the same structural motif. The observed frequency shifts and the differences in relative intensity are to be ascribed to interactional and/or orientational effects. To deepen the interpretation of the spectroscopic observations we performed a QM-based vibrational analysis of the title molecule and its corresponding gold adsorbate.

**Figure 2:**
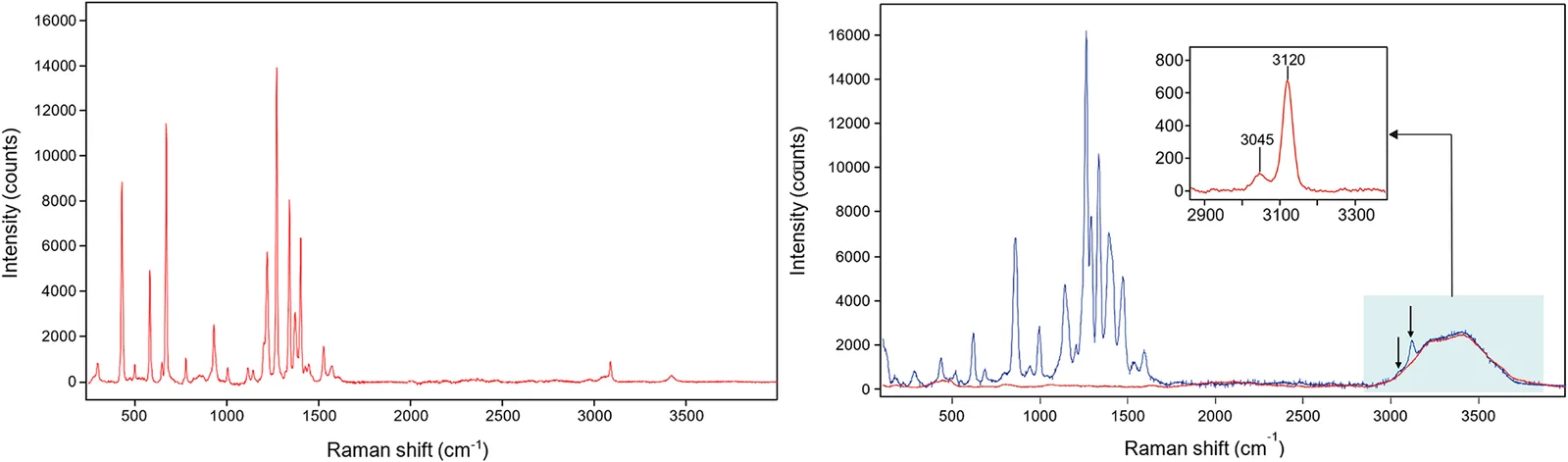
Left: spontaneous Raman spectrum of crystalline 6-MP. Right: SERS spectrum of a 20 μM aqueous solution of 6-MP (blue trace) and Raman spectrum of water (red trace). The inset displays the SERS spectrum of 6-MP in the 2880–3340 cm^–1^ interval, following removal of the solvent contribution.

### Vibrational Fingerprints of 6-MP Tautomers in Solution

The 6-mercaptopurine molecule can adopt several stable tautomeric forms (Figure 3), arising from the interplay of two distinct phenomena: (i) thione-thiol tautomerism, i.e., the equilibrium between the thione form and the thiol (6-purinethiol, in *cis* or *trans* conformations) form, and (ii) prototropic tautomerism, involving the migration of the labile proton among the available nitrogen donor sites of the purine ring (N_1_, N_3_, N_7_, N_9_). Consistently with previous studies,^77–80^ the thione tautomers Thione­(N_1_,N_7_) and Thione­(N_1_,N_9_) emerge as the most stable configurations in gas-phase and solution. To further refine our understanding of the stability of the tautomer in aqueous environments, we extended our analysis by incorporating an explicit/implicit hybrid solvation approach, where four water molecules explicitly interact with each tautomer (Figure S2) while the bulk solvent is modeled using C-PCM (Table 1), the detailed discussion is reported in Section S0.1.

**Figure 3:**
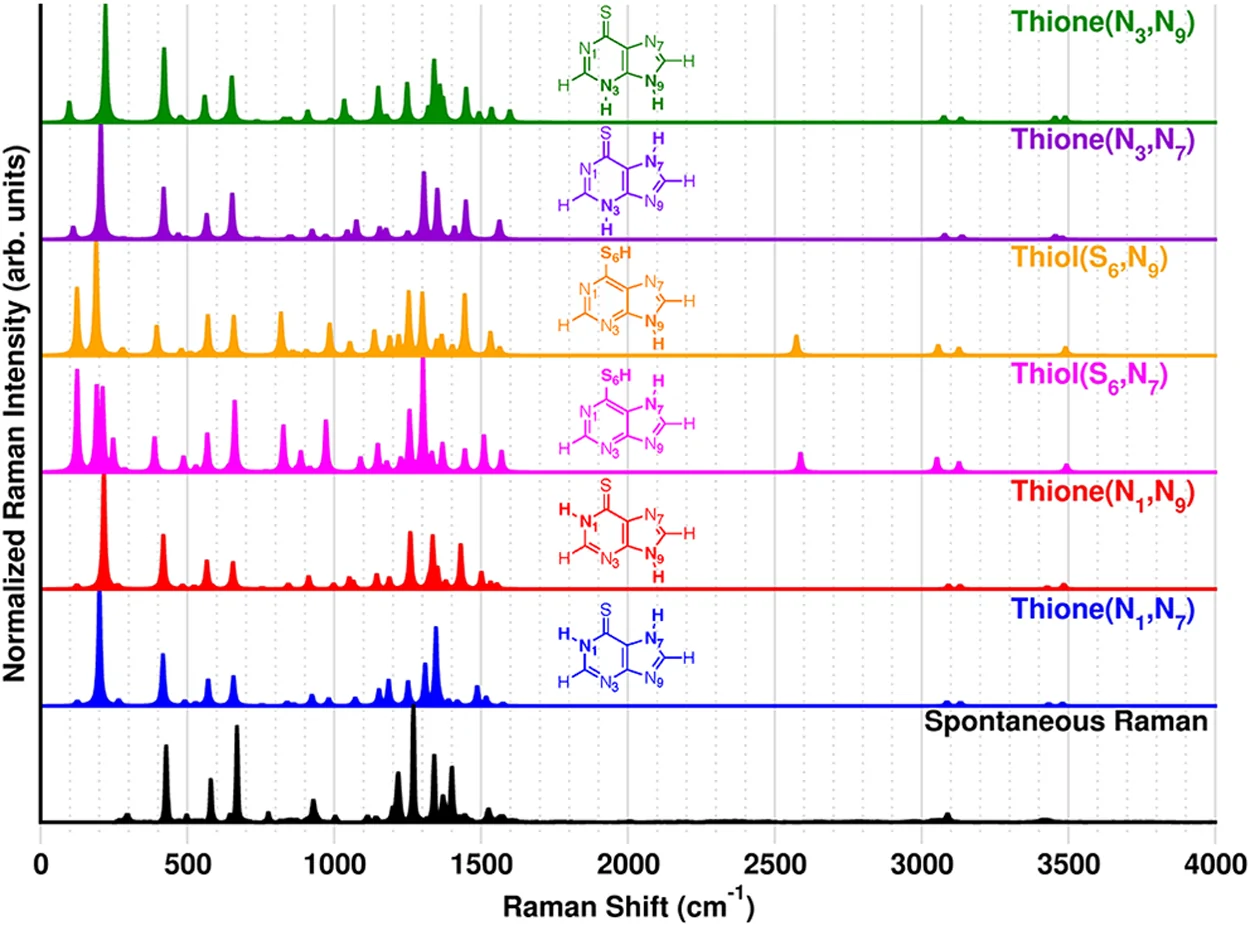
Normalized Raman spectra computed at the B3LYP/6–311+G­(d,p)/GD3BJ/C-PCM­(water) level of theory for all six tautomers of 6-mercaptopurine (6-MP), compared with the experimental spontaneous Raman spectrum reported at the bottom. Each spectrum is normalized to its own maximum peak intensity. The color legend for each tautomer is provided in the graph inset. Proton-bearing sites are reported in bold along with the numbering scheme adopted.

**Table 1:** Relative Electronic Energies (Δ*E*) and Gibbs Free Energies (Δ*G*) in kcal/mol for Different 6-Mercaptopurine Tautomers^a^

	Δ*E*_+ZPE_			Δ*G*		
	Gas	C-PCM	Cluster	Gas	C-PCM	Cluster
Thione(N_1_,N_7_)	0.00	0.00	0.13	0.00	0.00	0.06
Thione(N_1_,N_9_)	3.09	0.85	0.00	3.07	0.87	0.00
Thiol(S_6_,N_7_)	7.70	9.86	10.23	7.42	9.66	8.97
Thiol(S_6_,N_9_)	3.82	7.67	7.72	3.68	7.51	6.78
Thione(N_3_,N_7_)	8.32	5.70	2.70	8.21	5.64	2.51
Thione(N_3_,N_9_)	23.39	11.20	9.48	22.93	11.08	8.22

a Calculations were performed at the DFT level using the B3LYP/6-311+G­(d,p)/GD3BJ method in the gas phase, with the continuum solvation model (C-PCM), and within an hybrid implicit/explicit solvation cluster considering four water molecules as solvent. Zero-point energy (ZPE) corrections are included.

In this section we focus on the vibrational analysis of more stable tautomers: Thione­(N_1_,N_9_) and Thione­(N_1_,N_7_). The simulated Raman spectra of the six tautomers in C-PCM solution (Figure 3 and Table S2) have been compared with experimental one (Figure 2 and Table S10).

In detail, the experimental spectrum of 6-mercaptopurine can be divided into three main regions: the high frequency region (2000–3500 cm^–1^), the fingerprint region (1000–1600 cm^–1^) and the low-frequency region (within 1000 cm^–1^). In the first region, the intense bands around 2580, 3200, and 3500 cm^–1^ are attributed to localized stretching vibrations involving the S–H bond (in thiol forms) and the C–H and N–H bonds, respectively. The S–H stretching vibration lies in a spectrally uncrowded region and is clearly predicted for the thiol tautomers, whereas it is completely absent from the experimental spectrum. In the spontaneous Raman experiment, this missing S–H band (expected at ∼2580 cm^–1^ for thiol forms) indicates that thiol contributions are, at most, minor under the present low-hydration conditions. Accordingly, the experimental Raman pattern is primarily assigned to thione tautomers of 6-MP. In addition, Section S0.2 reports and discusses the computed optical absorption spectra of all six tautomers, which further support the coexistence of both Thione­(N_1_,N_7_) and Thione­(N_1_,N_9_) in solution, as suggested by the good agreement between the simulated and experimental UV–Vis profiles. The fingerprint region, is characterized by an intense band at 1269 cm^–1^ followed by signals of decreasing Raman activity at 1340, 1400, 1525, and 1570 cm^–1^. This spectral region is characteristic of aromatic heterocyclic compounds. The simulated spectrum of the Thione­(N_1_,N_9_) reproduces the experimental features reasonably well, both in terms of band profiles and relative peak positions. In particular, the calculated spectrum shows band at 1259, 1335, 1430, 1501, and 1533 cm^–1^ consistent in shape with the experimental ones. Some of the experimental signals can be however attributed to vibrational modes involving the Thione­(N_1_,N_7_) (Table S2 in SI), suggesting its participation in the overall experimental spectrum. At lower frequency, the experimental spectrum displays four prominent bands at 225, 427, 579, 668 cm^–1^, which are well reproduced by the Hessian-based Raman spectra of Thione­(N_1_,N_9_) (215, 417, 565, 655 cm^–1^) and Thione­(N_1_,N_7_) (200, 416, 570, and 656 cm^–1^) tautomers. The qualitative agreement between the experimental Raman spectrum and the computed vibrational fingerprints of Thione­(N_1_,N_7_) and Thione­(N_1_,N_9_) supports their assignment to the main observed features. This, in turn, suggests that both tautomers may plausibly coexist under the experimental conditions. To further explore this hypothesis and to account for additional vibrational bands observed in the experimental spectrum, we extended our analysis to include the Raman spectrum calculated from the crystallographic structures (reported below). The full comparative structural analysis is provided in Section S0.3.

### Raman Spectral Analysis of the X-ray-Based Dimer

In the solid state, 6-mercaptopurine is characterized by a pronounced structural diversity arising from prototropic tautomerism and variable hydration, as clearly evidenced by the two single-crystal X-ray structures considered here. The first form is a hemihydrate (0.5·H_2_O per 6-mercaptopurine), with two crystallographically independent 6-mercaptopurine molecules per water molecule, where the lattice is dominated by an extended N–H···N network and the water acts primarily as an O–H···S donor. The second form corresponds to a 1:1 monohydrate, in which one water molecule is incorporated per 6-mercaptopurine unit and participates directly in the supramolecular architecture through a bridging hydrogen-bond pattern that involves ring nitrogens and the thione sulfur. Importantly, the two crystals encode distinct prototropic tautomers differing in the location of the second ring proton (Thione­(N_1_,N_9_) and Thione­(N_1_,N_7_), respectively) and hydrogen bond topology while preserving the thione (C=S) form. In Figure 4 is reported a *vis-a-vis* comparison between the simulated and measured spontaneous Raman spectra. The vibrational assignments reported in Table 2 and Table S9 provide a detailed mapping between the experimental Raman spectrum of crystalline 6-MP and the harmonic spectra computed for the two crystal-derived dimer models (panels A and B in Figure 4). The spectrum computed for the crystal-derived dimer containing Thione­(N_1_,N_9_) tautomer and crystallization water molecules provides the closest match to experimental Raman profile, although the latter still include minor contributions that are compatible with Thione­(N_1_,N_7_), supporting the structural validity of the optimized X-ray-based dimers as proxies of the solid-state motif.

**Figure 4:**
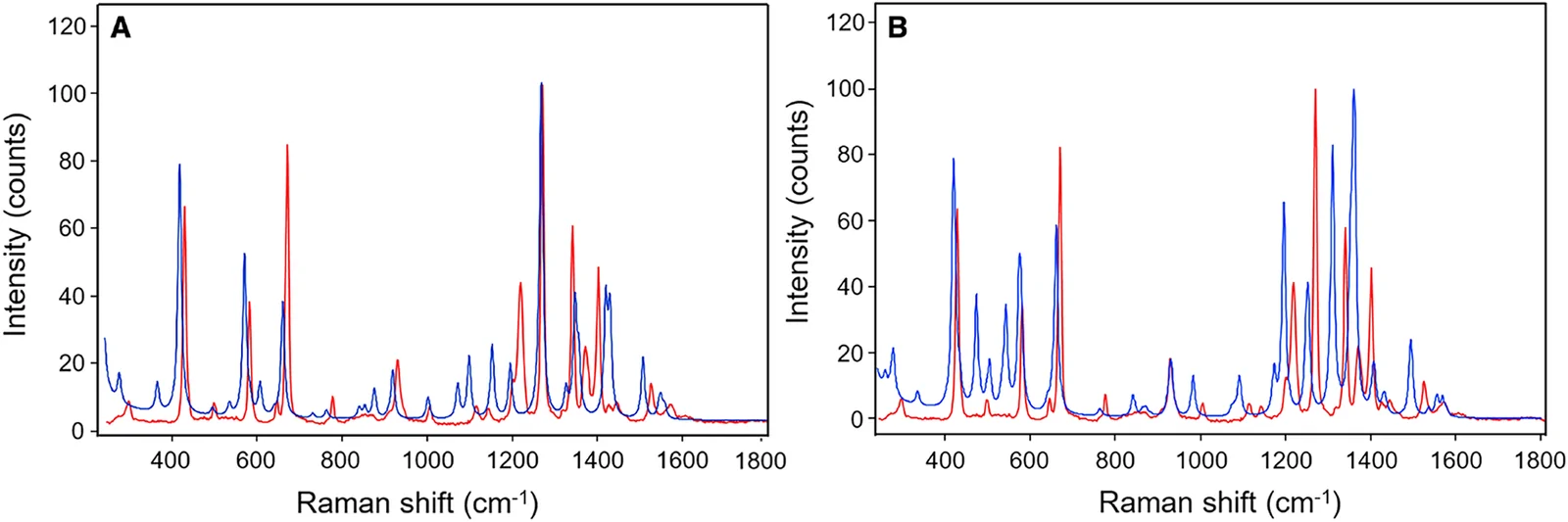
Comparison between the experimental spontaneous Raman spectrum of crystalline 6-MP (red trace) and the simulated Raman spectrum (blue trace) computed for dimeric motifs extracted from X-ray crystal structures and subsequently optimized at the DFT level. Panel A: dimer model derived from the crystal structure shown in Figure S4. Panel B: dimer model derived from the crystal structure shown in Figure S5.

**Table 2:** Comparison between Observed and Calculated Raman Bands (Figure 4, Left Side), with Relative Normalized Intensities in Parentheses, Percentage Error, and Vibrational Assignments^a^

Obs. (cm^–1^)	Calc. (cm^–1^)	Err. (%)	Description
296 (0.06)	273 (0.14)	7.8	Out-of-plane def pr
–	363 (0.11)		ω(H_2_O)
427 (0.64)	415 (0.76)	2.8	In-plane def r1
497 (0.06)	493 (0.04)	0.8	In-plane def pr
540 (0.02)	533 (0.06)	1.3	Out-of-plane def pr
579 (0.36)	568 (0.49)	1.9	In-plane def pr
–	605 (0.12)		ω(H_2_O)
645 (0.06)	638 (0.05)	1.1	ω(NCNH)r2
668 (0.82)	658 (0.35)	1.5	In-plane def pr
775 (0.08)	762 (0.03)	1.7	Out-of-plane def r1/Out-of-plane def pr
–	874 (0.10)		ω(CNCH)r2
912 (sh, 0.02)	907 (sh, 0.04)	0.5	ω(CH)r1
928 (0.19)	917 (0.15)	1.2	In-plane def r2/ω(NCNH)r1/ω(CNCH)r1
1003 (0.05)	1000 (0.07)	0.3	In-plane def pr/ν(CS)
–	1071 (0.11)		δ(NCH)r2/δ(CNH)r2
1114 (0.05)	1097 (0.19)	1.5	ν(CN)r1/δ(CNH)r2
1142 (0.04)	1151 (0.23)	0.8	ν(CN)r1/ν(NC)r1
1198 (0.13)	–		In-plane def r1/ν(CS)r2/δ(NCH)r2
1218 (0.42)	1194 (0.17)	2.0	ν(NC)r2/ν(CN)r2/δ(NCH)r2
1269 (1)	1266 (1)	0.2	ν(NC)r2/δ(NCH)r2/δ(CNH)r2
1317 (0.04)	1325 (0.11)	0.6	ν(NC)r1/δ(NCH)r1
1340 (0.58)	1346 (0.38)	0.4	δ(HNC)r1/δ(HCN)r1
1370 (0.23)	1356 (0.24)	1.0	ν(CN)r1/ν(CC)r1/ν(CN)r2/ν(CC)r1
1377 (0.15)	–		
1400 (0.46)	1418 (0.40)	1.3	δ(CNH)r2/δ(NCH)r1
1426 (0.05)	1428 (0.37)	0.1	ν(NC)r2/δ(HCN)r2/δ(HNC)r2
1443 (0.06)	–		
1525 (0.12)	1506 (0.19)	1.2	δ(CNH)r1
1539 (0.02)	–		
1558 (0.04)	1549 (0.08)	0.6	δ(H_2_O)
1570 (0.05)	1558 (0.05)	0.8	In-plane def r1/δ(H_2_O)
$\overset{®}{\mathrm{Err}}=1.1\pm 0.6$			

a pr = purine structure; ring 1 (r1) and ring 2 (r2) are identified in Figure 1, left side; ν = stretching; δ = bending; ω = wagging; the notation of wagging modes is Atoms 1 and 3 define the ends of the four-atom fragment. Atom 2 is the central atom, and atom 4 is the substituent bonded to atom 2. The wagging mode corresponds to an out-of-plane motion of atom 4 relative to the plane formed by atoms 1–2–3.

In Figure 4, panel A, the experimental spectrum is compared with the one simulated for dimer containing Thione­(N_1_,N_9_), reported in Figure S4. A band-by-band correlation between observed and predicted features, together with the corresponding vibrational assignments, is reported in Table 2. The lowest frequency experimental band at 296 cm^–1^ is reproduced at 273 cm^–1^ (7.8% deviation). This relatively large discrepancy is not unexpected, since low-frequency, lattice-like motions in this spectral region are more reliably captured by fully periodic approaches.^81^ Upon excluding this feature, the agreement becomes excellent, with average deviation of 1.1 ± 0.6%, well within the typical accuracy of DFT Raman simulations for molecular systems (∼2%).^82^ In particular, the characteristic low-frequency thione features observed at 427, 579, 668, and 928 cm^–1^ are reproduced at 415, 568, 658, and 917 cm^–1^, respectively. Most importantly, the computed spectrum captures the diagnostic fingerprint pattern (exp. 1003, 1269, 1317, 1340 cm^–1^ vs calc. 1000, 1266, 1325, 1346 cm^–1^): the most intense experimental band at 1269 cm^–1^ is reproduced as the dominant calculated peak at 1266 cm^–1^ with an error of only 0.2%, and the neighboring bands in the 1200–1600 cm^–1^ region are recovered with the correct ordering and relative intensity trend. Additional agreement is found for key markers such as the feature at 1340 cm^–1^ (computed at 1346 cm^–1^) and the band at 1003 cm^–1^ (computed at 1000 cm^–1^), further supporting Thione­(N_1_,N_9_) dimer as the most representative crystal-based model. Beyond peak positions, inspection of Figure 4 and Table 2 indicates that the calculation also provides a reasonable description of relative Raman intensities. Two medium intensity computed bands at 363 and 605 cm^–1^ are not observed experimentally. Both correspond to localized wagging modes of the crystallization water molecules suggesting that small uncertainties in the experimental placement of the water molecules and/or limitations of harmonic approximation may affect their predicted Raman activity. The agreement is significantly lower when considering the Thione­(N_1_,N_7_) dimer (Figure S5), with larger deviations in both peak positions and relative intensities, see Table S9 in SI, highlighting the sensitivity of the calculated spectrum to subtle differences in hydrogen bonding topology and supramolecular arrangement.

### Effect of Gold Substrates on Structure and Raman Response of 6-MP Tautomers

To investigate any structural and spectroscopic effects deriving from the interactions between 6-MP and gold substrates, we initially selected two cluster models—Au_6_ and Au_20_—in combination with the most populated/stable tautomers, Thione­(N_1_,N_7_) and Thione­(N_1_,N_9_). The species optimized in C-PCM are taken as a reference and the calculated Raman spectra of both tautomers are reported in Figure 5. The present calculations address the chemical contribution to SERS (CM), i.e., adsorption-induced changes in polarizability and selection rules, while the electromagnetic enhancement (EM) is not explicitly modeled and is treated only qualitatively. Therefore, our discussion focuses on relative band patterns and adsorption-sensitive markers, rather than absolute enhancement factors.

**Figure 5:**
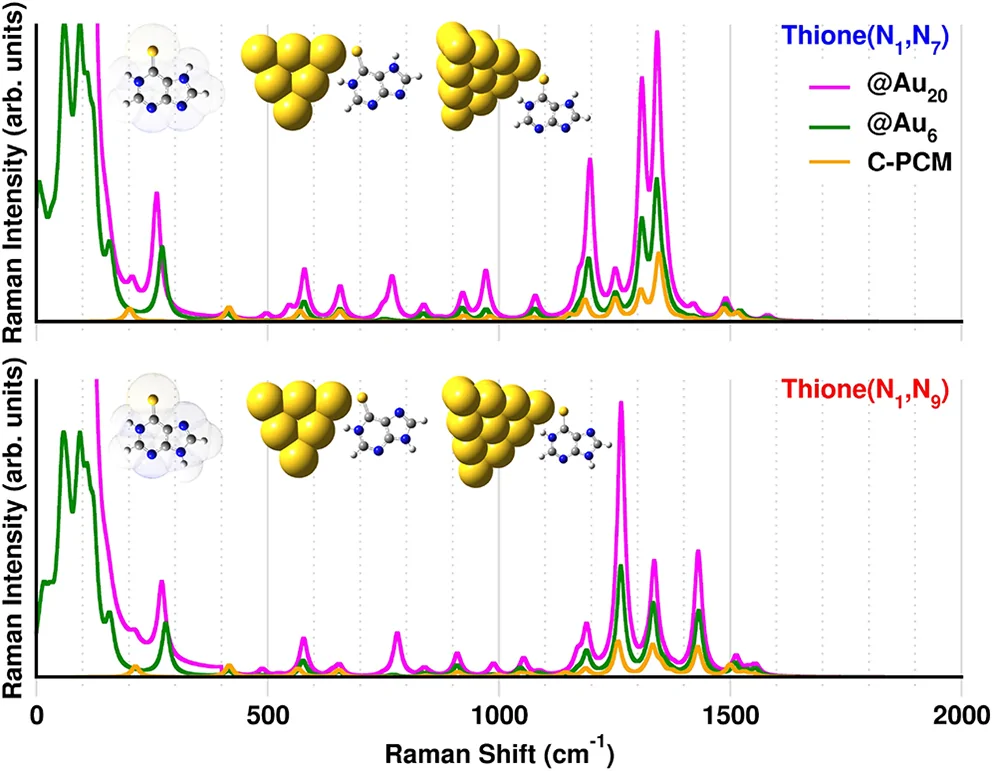
Raman spectra of Thione­(N_1_,N_7_), and Thione­(N_1_,N_9_), computed in C-PCM, adsorbed on Au_6_ and Au_20_ clusters. Atomic radii are scaled for visual clarity.

These models provide a convenient representation of the local chemical environment of the gold surface relevant for the chemical contribution to the SERS effect, as discussed in literature.^42^ It should be noted, however, that these models are not designed to reproduce the plasmonic response of extended gold surfaces. Upon adsorption of 6-MP on the gold clusters, the C–S bond undergoes a slight elongation of 0.023 Å (from 1.683 Å in the isolated molecule computed in C-PCM). A similar effect is observed for the N–H bond oriented toward the gold surface, which increases from 1.014 Å to 1.024 Å for both tautomeric species. By contrast, the remaining geometry, particularly the five-membered ring, is only marginally affected, with negligible structural changes observed in both tautomers. The Raman spectrum remains largely unchanged across the models, indicating that the local coordination does not significantly perturb the vibrational frequencies. A particularly relevant result is the appearance of a signal attributable to the out-of-plane of N_1_–H, 767 (Au_6_) and 769 (Au_20_) cm^–1^ for the complexes with Thione­(N_1_,N_7_) and 786 (Au_6_) and 780 (Au_20_) cm^–1^ for those with Thione­(N_1_,N_9_). Therefore, a clear trend emerges in terms of Raman activity, as it becomes progressively more intense as the size of the gold nanocluster increases. Variations in Raman activity can be interpreted as an intrinsic signature of the chemical effect induced by the metal, that is, of the changes in the molecular electronic structure and vibrational polarizability brought about by adsorption. Binding energy calculations (Table 3) confirm the increased stabilization upon adsorption, with the Thione­(N_1_,N_9_) species consistently exhibiting stronger binding to both Au_6_ and Au_20_ clusters compared to Thione­(N_1_,N_7_).

**Table 3:** Total and Per-Atom Binding Free Energies (Δ*G*_bind_) in kcal/mol for the Thione­(N_1_,N_7_) and Thione­(N_1_,N_9_) Complexes with Au_6_ and Au_20_ Clusters^a^

Adduct	Δ*G*_bind_	Δ*G*_bind_^norm^
Thione(N_1_,N_7_)@Au_20_	–8.00	–0.40
Thione(N_1_,N_9_)@Au_20_	–10.35	–0.52
Thione(N_1_,N_7_)@Au_6_	–11.85	–1.98
Thione(N_1_,N_9_)@Au_6_	–11.94	–1.99

a The total binding free energy is computed as Δ*G*_bind_ = *G*_complex_ – (*G*_6–MP_ + *G*_cluster_), and converted from Hartree to kcal/mol using a factor of 627.509. The per-atom quantity is obtained by normalizing over the number of gold atoms: $\Delta G_{\mathrm{bind}}^{\mathrm{norm}}=\frac{\Delta G_{\mathrm{bind}}}{n_{\mathrm{Au}}}$.

In the following, we focus on the 6-MP@Aur_20e_ sytems, which represent a reasonable compromise between structural realism and computational cost. Indeed, to simulate the surface of gold nanoparticles, the use of tetrahedral Au_20_ cluster, already used in several theoretical studies,^42,83–86^ was expected to provide more reliable results than the smaller size Au_6_. This model, although does not correspond to experimentally known clusters,^87–89^ allowed us to study the interaction of the 6-MP with the surface Au atoms, therefore to elucidate how the local different environments may affect the spectroscopic properties of 6-MP. The Au_20_ cluster adopts a tetrahedral (T_d_) geometry, corresponding to a structurally relaxed fragment of the face-centered cubic lattice of bulk gold exposing (111)-like facets. This makes it a unique model system, as it combines atomic packing reminiscent of the extended metal surface with distinct molecular properties.^90–93^

### Adsorption Motifs of 6-MP on Au_20_ Nanocluster

The tetrahedral structure of Au_20_ allows for multiple adsorption geometries of both Thione­(N_1_,N_7_) and Thione­(N_1_,N_9_) species. Among the investigated configurations, we considered three representative cases: two configurations (6-MP on *vertex*, and 6-MP in the *center*) are formed by anchoring the sulfur atom on a gold atom, whereas in the third (6-MP *flat*) the 6-MP takes an orientation parallel to the gold surface, see Figure 6.

**Figure 6:**
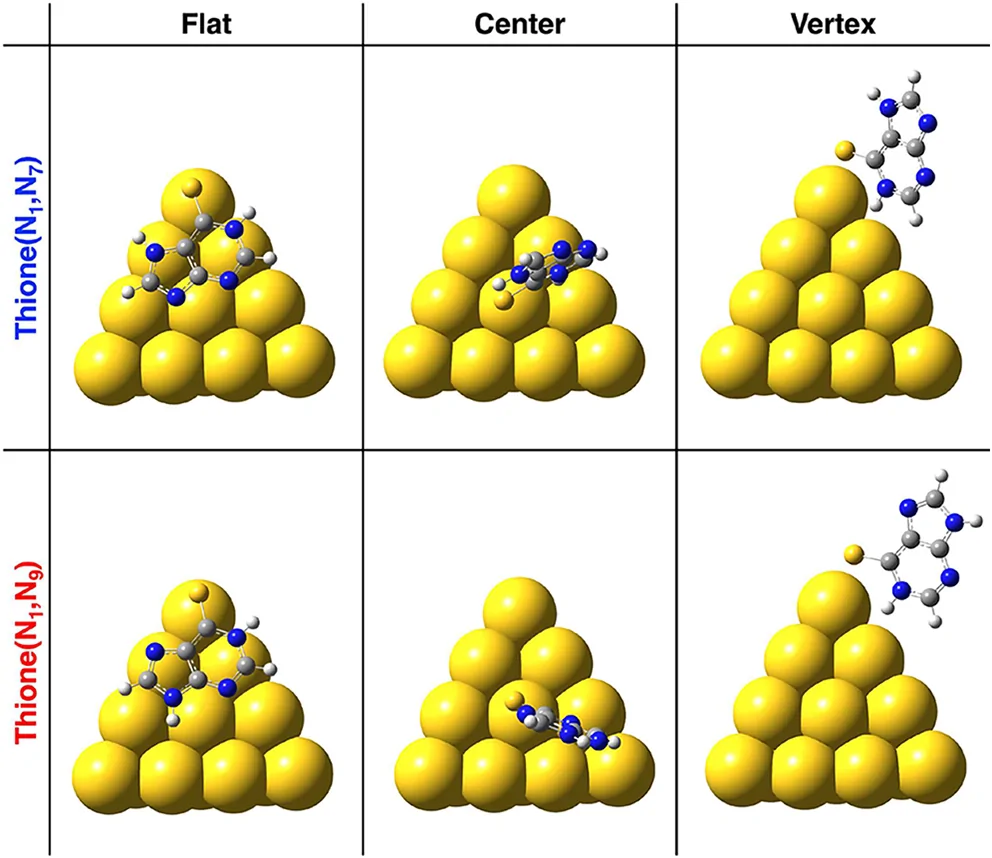
Optimized structures of Thione­(N_1_,N_7_) and Thione­(N_1_,N_9_) tautomers adsorbed on different sites of the Au_20_ cluster, computed at B3LYP/6–311+G­(d,p)/GD3BJ/C-PCM level of theory. For each system, three adsorption sites are considered: “*flat*”, “*center*”, and “*vertex*” of the Au_20_ cluster. Atomic radii are scaled for visual clarity. Refer to Table 4 for the binding energies.

Briefly, for the *flat* configuration, both tautomers exhibit a very similar structural arrangement, with an S–Au distance of 2.70 Å for Thione­(N_1_,N_7_) (2.67 Å for Thione­(N_1_,N_9_)), and lie parallel to the surface of Au_20_ nanocluster. This is further confirmed by the C–S–Au angle, which is 91.72° for Thione­(N_1_,N_7_) (89.74° for Thione­(N_1_,N_9_)). In the *center* configuration, 6-MP has the sulfur atom oriented toward the Au atom located at the center of the hexagonal facet on the Au_20_ cluster, with N_1_–H bond also pointing toward the surface. For Thione­(N_1_,N_7_), the S–Au distances remain similar to the previous configurations. We anticipate that the orientation of the N_1_–H bond toward the gold surface will prove to be a key factor in the fine interpretation of the experimental SERS spectrum. In the configuration referred to as *vertex*, the 6-MP backbone is aligned along one of the edges of the Au_20_ cluster, with the sulfur atom pointing toward the gold atom located at the apex of the tetrahedron (at ∼2.54 Å in both cases). The N_1_–H bond is also oriented toward the gold surface, similarly to what is observed in the *center* configuration. As shown in Table 4, the binding energies are significantly negative and thus the adsorption processes are spontaneous. In particular, the evaluation of the thermodynamic stability of the 6-MP@Au_20_ complexes via the binding energies leads to the conclusion that the most stable complex is the 6-MP *flat* configuration (−17.63 and −17.07 kcal/mol for Thione­(N_1_,N_9_) and Thione­(N_1_,N_7_), respectively). The other two configurations have very similar binding energies: −10.41 and −10.66 kcal/mol for Thione­(N_1_,N_9_)@Au_20_ and −8.41 and −7.91 kcal/mol for Thione­(N_1_,N_7_)@Au_20_ on *vertex* and in the *center*. Notably, the adsorption motif that best reproduces the experimental SERS pattern does not necessarily coincide with the global thermodynamic minimum, because real SERS substrates expose a heterogeneous surface landscape in which multiple adsorption microenvironments can coexist.^23,94^ However, a careful analysis of the calculated Raman spectra leads to a different conclusion, namely the complex Thione­(N_1_,N_9_)@Au_20_ combined with that of Thione­(N_1_,N_7_)@Au_20_, both in *vertex* configurations, produces a spectrum very similar to the experimental one. Indeed, as shown in the spectra in Figure S6, the Thione­(N_1_,N_9_)@Au_20_ and Thione­(N_1_,N7)@Au_20_ in *flat* configuration exhibit a distinct/different vibrational pattern (in terms of peak positions and Raman activities) compared to the experimental SERS spectrum (see Table S10). Notably, we observe the presence of one dominant peak at 577 cm^–1^ for the Thione­(N_1_,N_9_) and at 572, 575 cm^–1^ for the Thione­(N_1_,N_7_), which are absent in the experimental spectrum and assigned to the out-of-plane deformation involving both rings of purine scaffold. In contrast, for the Thione­(N_1_,N_9_)@Au_20_ the spectra of the other two clusters configurations (*center* and *vertex*, in Figure 7) have a signal pattern comparable with that of the experimental SERS one and similar to each other.

**Table 4:** Binding Free Energies, Δ*G*_bind_ (kcal/mol), for the Thione Tautomers Adsorbed on Different Sites of the Au_20_ Cluster, Computed at B3LYP/6-311+G­(d,p)/GD3BJ/C-PCM Level of Theory^a^

Δ*G*_bind_	Flat	Center	Vertex
Thione(N_1_,N_7_)@Au_20_	–17.07	–7.91	–8.41
Thione(N_1_,N_9_)@Au_20_	–17.63	–10.66	–10.41

a For each system, three adsorption sites are considered: “*flat*”, “*center*”, and “*vertex*” of the Au_20_ cluster. Refer to Figure 6 for structures.

**Figure 7:**
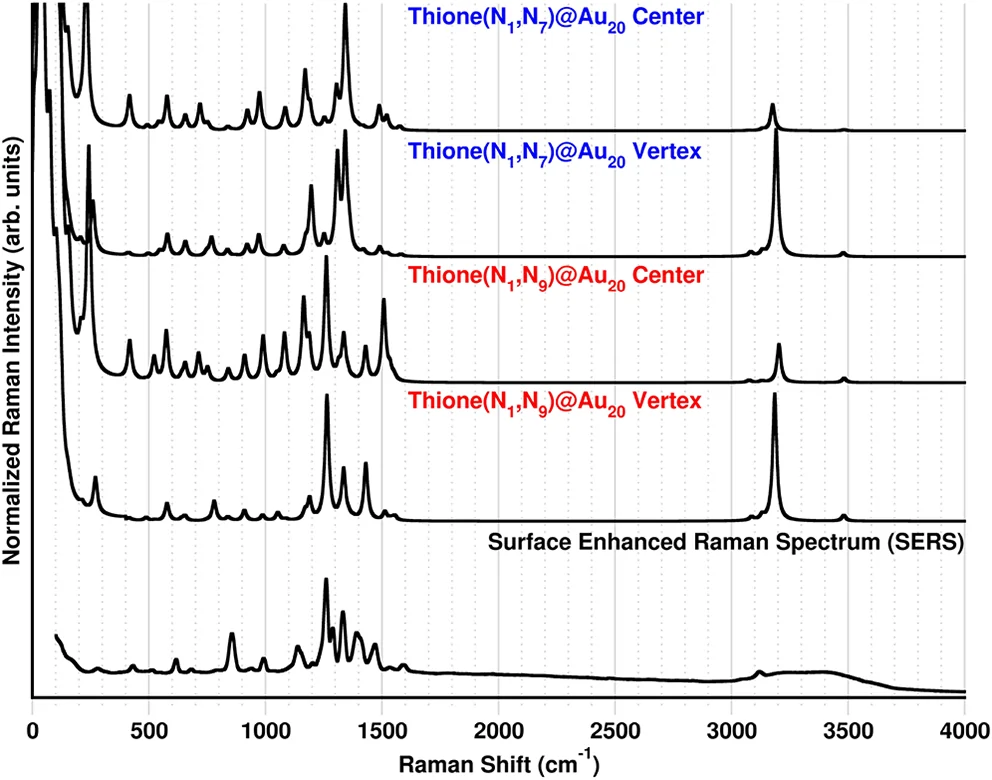
Normalized Raman spectra of Thione­(N_1_,N_7_) and Thione­(N_1_,N_9_) tautomers adsorbed on Au_20_ cluster in *center* and *vertex* configurations computed at B3LYP/6–311+G­(d,p)/GD3BJ/C-PCM level of theory. The experimental SERS spectrum is reported at the bottom for comparison.

In detail, the high frequency region (3000–3500 cm^–1^), we observe signals arising from C–H and N–H stretching vibrations. The fingerprint region of both computed spectra is characterized by two little peaks at 1188 and 1189 cm^–1^ and by five signals of decreasing Raman activity at ∼ 1264, 1335, 1430, 1513, and 1555 cm^–1^ which correspond to the experimental peaks at 1140, 1261, 1333, 1410, 1533, and 1593 cm^–1^. In the low-frequency region (within 1000 cm^–1^), a peak at 780 cm^–1^ corresponding to the out-of-plane vibration of N_1_–H group is observed only in the spectrum of Thione­(N_1_,N_9_)@Au_20_ in the *vertex* configuration, matching the 850 cm^–1^ peak in the experimental SERS spectrum. The presence of this feature, absent in the spontaneous Raman spectrum, is indicative of a significant interaction between the 6-MP and the Au surface, which strongly depends on the relative orientation of Thione­(N_1_,N_9_) with respect to the Au_20_ cluster. Similarly, the spectra of the two configurations of Thione­(N_1_,N_7_)@Au_20_, one on the *vertex* and the other one on the *center*, show high frequency spectral features related to the C–H and N–H stretching modes. However, the out-of-plane N_1_–H at 769 cm^–1^ appears only in the *vertex* configuration. In the fingerprint region the Thione­(N_1_,N_9_)@Au_20_ spectrum exhibits signals at 1172, 1197, 1252, 1309, 1342 (a shoulder at 1360), 1490 and 1523 cm^–1^ which seem to complete the pattern of signals present in the SERS experimental spectrum. Based on these observations, we hypothesize that both tautomers, Thione­(N_1_,N_7_) and Thione­(N_1_,N_9_), are present on the gold surface in a *vertex* configuration. In the following sections, we will explore clusters containing up to 4 molecules on the same gold nanocluster.

### Multitautomer Adsorption Models and SERS Interpretation

According to these results, it is evident that the 6-MP molecules are bonded to the Au surface through the S atoms with a nearly vertical orientation. In this configuration, the aromatic rings of the 6-MP should be arranged in parallel with each other contributing to stabilize the interaction between the drug and the Au surface. One of the tasks for the study of these systems is deciding how to characterize all the effects. In this work we face this challenge for the first time. To explore this situation, initially, we selected four different clusters with the Au_20_ pyramid (Figure 8) and one (A), two (A,B), three (A,B,C) and four (A,B,C,D) 6-MP Thione­(N_1_,N_9_) molecules, respectively.

**Figure 8:**
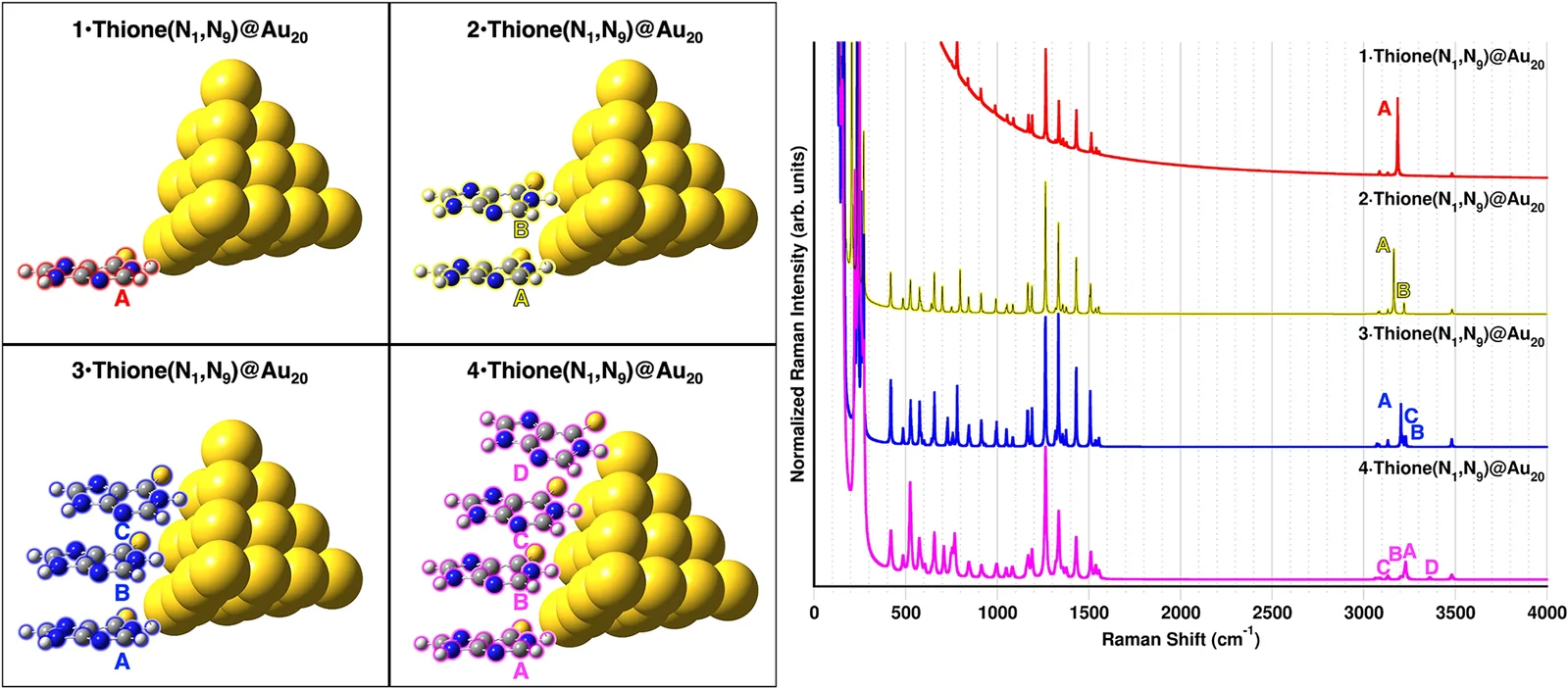
Left: Optimized structures of Thione­(N_1_,N_9_)@Au_20_ complexes containing one to four adsorbed 6-mercaptopurine units computed at the B3LYP/6–311+G­(d,p)/GD3BJ/C-PCM level of theory. The binding free energies are reported in Table 5. Right: corresponding normalized Raman spectra of Thione­(N_1_,N_9_)@Au_20_ complexes with increasing number of adsorbed 6-mercaptopurine units at an incident wavelength of 638 nm. See also Figure S7.

As shown in Table 5, the binding energies of the four Thione­(N_1_,N_9_) clusters become more negative (by 2 kcal/mol) as 6-MP molecules increase and this is likely to depend on an energy gain due to the π-π interactions between aromatic rings.

**Table 5:** Binding Free Energies, Δ*G*_bind_ (kcal/mol), for Thione­(N_1_,N_9_)@Au_20_ Models Containing One to Four Adsorbed 6-Mercaptopurine Units (*n* = 1–4), Computed at the B3LYP/6-311+G­(d,p)/GD3BJ/C-PCM Level of Theory

6-MP units (*n*)	Δ*G*_bind_
1·Thione(N_1_,N_9_)@Au_20_	–10.41
2 1·Thione(N_1_,N_9_)@Au_20_	–12.14
3 1·Thione(N_1_,N_9_)@Au_20_	–12.73
4 1·Thione(N_1_,N_9_)@Au_20_	–12.56

Moreover, a detailed analysis of the calculated Raman spectra (right panel of Figure 8 and Table 6) reveals a progressive enhancement of signal intensities as additional 6-MP units are coordinated to the gold nanocluster but with an almost unchanged pattern except for the signals related to out-of-plane and stretching of N_1_–H. Regarding the out-of-plane signal of N_1_–H as the number of mercaptopurines increases the peak slightly widens: 780 cm^–1^ with one, 728 and 797 cm^–1^ with two 6-MP molecules, 725, 729, and 781 cm^–1^ with three and 708, 767, 768, and 773 cm^–1^ with four. This vibrational signal also exhibits contributions from collective motions involving both aromatic rings of the adsorbed 6-MP units. As the number of coordinated molecules increases, the coupling between adjacent units enhances the complexity of the vibrational pattern, leading to the observed spectral broadening and mode splitting. This is in accordance with the fact that each of these hydrogen atoms suffers from slightly different effects around. Observing signals in the high frequency region of the smallest cluster, Thione­(N_1_,N_9_)@Au_20_ on *vertex*, the N_1_–H stretching has a very strong signal, 3185 cm^–1^ (A red spectrum in Figure 8), this result is not in agreement with the experimental data, where N_1_–H signals are much less intense than those of the fingerprint region. On the other hand, as the number of 6-MP increases on the gold surface, a noticeable attenuation in the intensity of this peak—labeled A—is observed (3164, 3203, and 3228 cm^–1^) in association with a distribution of the signals relating to the different molecules (B at 3220 cm^–1^ yellow spectrum; B and C at 3230 and 3214 cm^–1^ blue spectrum; and B, C, and, D at 3221, 3203, 3361 cm^–1^ magenta spectrum). These data would fully explain the presence of very weak signals in the experimental SERS spectrum. It is thus possible to suggest that the molecules of 6-MP are bound through the sulfur atom to the gold surface and are placed in parallel with each other. It should be noted that the signals most representative of the experimental situation are those relating to the central molecules (B and C). This is because our model represents a small portion of a very large surface with a high number of molecules and consequently the effect due to the surrounding molecules is better represented with the inner molecules.

**Table 6:** Computed Raman Peak Positions (in cm^–1^) for 6-Mercaptopurine Molecules Adsorbed on Au_20_ Clusters, Considering Increasing Surface Coverage from One to Four Thione­(N_1_,N_9_) Units. The experimental SERS spectrum is provided in the last column for comparison^a^

1·Thione(N_1_,N_9_)@Au_20_	2·Thione(N_1_,N_9_)@Au_20_	3·Thione(N_1_,N_9_)@Au_20_	4·Thione(N_1_,N_9_)@Au_20_	SERS exp.
780	728	725	708	850
	797	729	767	
		781	768	
			773	
1170/1190	1164/1188	1161/1188	1159/1188	1140
	1167/1189	1164/1189	1165/1189	
		1167/1190	1166/1190	
			1169/1191	
1264	1262	1260	1260	1261
	1263	1261	1262	
		1263	1263	
			1264	
1335	1333	1332	1332	1333
	1334	1333	1334	
		1334	1335	
			1336	
1430	1429	1429	1428	1410
	1431	1430	1429	
		1431	1430	
			1431	
1513	1504	1503	1506	1470
	1509	1506	1509	
		1507	1510	
			1511	
1539	1534	1532	1531	1533
	1538	1534	1534	
		1537	1537	
			1538	
1555	1552	1550	1552	1593
	1554	1552	1553	
		1555	1554	
			1555	
3185 (A)	3164 (A)	3203 (A)	3203 (C)	//
	3220 (B)	3214 (C)	3221 (B)	
		3230 (B)	3228 (A)	
			3361 (D)	

a Labels (A–D) indicate specific geometric configurations corresponding to distinct adsorption motifs used in the calculation of the tetrameric system.

As anticipated, we assume that on the gold surface of nanoparticles there are both Thione­(N_1_,N_9_) and Thione­(N_1_,N_7_). So far, in the literature there are no studies where gold clusters with more than one tautomeric form are considered. In this work, we analyze clusters with both Thione­(N_1_,N_9_) and Thione­(N_1_,N_7_). Therefore, we have selected a series of clusters with different stoichiometric ratios of the two tautomers such as 1:1, 2:1, 3:1, and, 2:2 in different combinations. From a careful comparison of the computed Raman spectrum with the experimental SERS one, we found that the main vibrational features of all clusters considered—within ∼1600 cm^–1^—show almost the same vibrational frequencies. On the contrary, the relative Raman intensity of the peaks in the fingerprint region is better reproduced by the 3:1 ratio model. Consequently, the most representative model was found to be that with 3:1 ratio (see the left panel of Figure 9 and Table 7). In detail, for the cluster with three Thione­(N_1_,N_9_) and one Thione­(N_1_,N_7_) we have selected four combinations which differ from each other for the relative adsorption site of Thione­(N_1_,N_7_). The right panel of Figure 9 shows the calculated Raman spectra for the four models and, as you can see, the signals are almost overlapping with the experimental ones both in the fingerprint and in the out-of- plane of the N_1_–H (between 680 and 780 cm^–1^) region. As mentioned in the previous paragraph, the most representative spectra are those associated with models B and C, in which the influence of surrounding molecules is more realistically captured, better reflecting the actual behavior of the real system.

**Figure 9:**
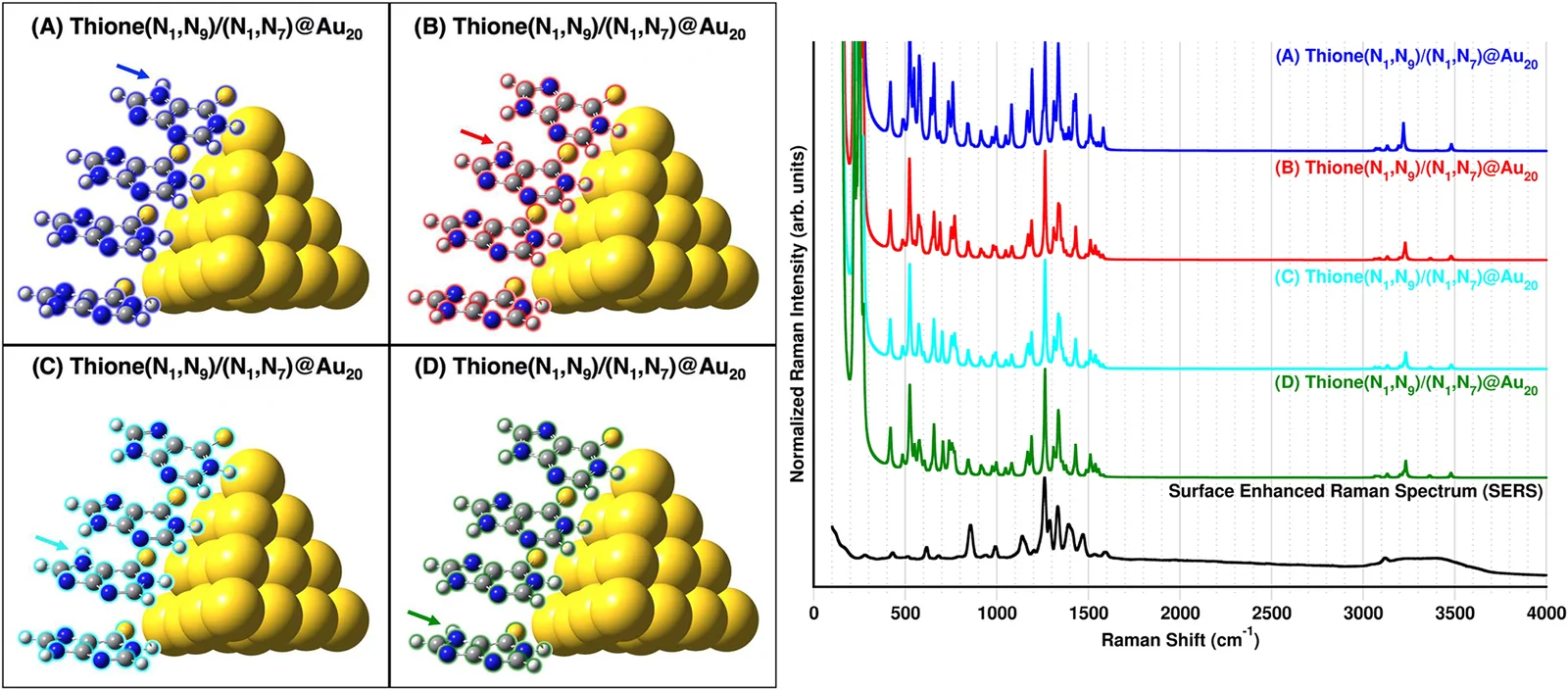
Left: Optimized structures of Thione­(N_1_,N_9_)/(N_1_,N_7_)@Au_20_ complexes containing adsorbed 6-mercaptopurine units in a 3:1 ratio computed at the B3LYP/6–311+G­(d,p)/GD3BJ/C-PCM level of theory. The arrow shows the Thione­(N_1_,N_7_) site. Right: corresponding normalized Raman spectra of the same complexes, at an incident wavelength of 638 nm, compared to the experimental SERS spectrum (black trace). See also Figure S10.

**Table 7:** Computed Raman Peak Positions (in cm^–1^) for Thione­(N_1_,N_9_)/(N_1_,N_7_) in a 3:1 Ratio Adsorbed on Au_20_ Clusters^a^

(A) Thione(N_1_,N_9_)/(N_1_,N_7_)@Au_20_	(B) Thione(N_1_,N_9_)/(N_1_,N_7_)@Au_20_	(C) Thione(N_1_,N_9_)/(N_1_,N_7_)@Au_20_	(D) Thione(N_1_,N_9_)/(N_1_,N_7_)@Au_20_	SERS exp.
687	690	701	705	850
734	769	763	739	
740	770	770	769	
776	775	781	770	
1162/1189	1159/1188	1159/1188	1159/1188	1140
1164/1190	1166/1189	1165/1189	1164/1189	
1166/1191	1168/1190	1167/1190	1167/1189	
1168/1193	1171/1191	1172/1191	1169/1190	
1249	1250	1251	1250	1261
1260	1261	1260	1260	
1262	1262	1263	1261	
1264	1263	1264	1263	
1333	1332	1332	1332	1333
1334	1335	1335	1334	
1335	1335	1336	1335	
1340	1343	1344	1340	
1375	1375	1375	1376	1392
1375	1376	1376	1376	
1377	1377	1377	1377	
1393	1393	1394	1390	
1418	1430	1418	1418	1470
1429	1430	1428	1428	
1430	1431	1430	1430	
1431	1487	1431	1430	
1505	1508	1521	1531	1533
1507	1511	1532	1534	
1509	1511	1537	1534	
1522	1522	1539	1538	
1552	1553	1552	1552	1593
1553	1555	1555	1554	
1555	1556	1555	1555	
1581	1577	1578	1579	
3194	3201	3202	3203	//
3218	3222	3220	3219	
3221	3230	3233	3233	
3400	3367	3365	3363	

a The experimental SERS peaks are provided in the last column for comparison. See also Figure S10.

As a representative example, we discuss here the main vibrational modes in terms of their composition—see Figure S12—and displacement patterns, in order to provide a more comprehensive interpretation of the SERS spectrum based not only on the frequency positions but also on the internal structure of the modes. For this purpose, we focus on the cluster labeled as B (Figure 8) and, to simplify the analysis, we restrict our discussion to a single 6-MP unit. Preliminary analysis confirms that the vectorial composition of the vibrational modes is essentially identical across the four molecular subunits, thus validating this simplification without loss of generality. The vibrational mode centered at 775 cm^–1^ is primarily associated with an out-of-plane bending of the N–H group directed toward the gold nanocluster. At 1166 cm^–1^, the mode consists of a delocalized in-plane deformation across both purine rings, dominated by C–N stretching with minor C–H bending contributions, characteristic of the skeletal movements of conjugated heterocycles. The 1189 cm^–1^ mode involves in-plane C–H bending and C–N stretching, mainly localized on the purine ring but with partial delocalization through the molecular scaffold. The vibration at 1261 cm^–1^ arises from an in-plane N–H bending motion coupled with C–N and C–C stretching, contributing notably to the Raman fingerprint region due to its mixed character and significant polarizability change. The 1335 cm^–1^ mode features delocalized C–N and C–C stretching across the purine core, representing a typical marker of the heterocyclic backbone. At 1377 cm^–1^, the motion is dominated by synchronized C–N and C–C stretching within the purine framework, with additional in-plane N–H bending components. The 1430 cm^–1^ mode includes a mix of in-plane bending and stretching, involving N–H and C–N bonds, with ancillary C–H deformation in the aromatic system. The 1508 cm^–1^ signal is primarily defined by C–C stretching adjacent to the sulfur atom and a simultaneous in-plane bending of the NH group. At 1555 cm^–1^, a strong C=C and C–N stretching character is observed within the six-membered ring. Finally, the high-frequency mode at 3222 cm^–1^ corresponds to a localized N–H stretching vibration, again oriented toward the gold surface.

The calculated spectra of models with ratios 1:1, 2:1 and 2:2 are shown in Figures S8, S9, and S11 in SI. In conclusion, the simulated Raman spectrum of the model with three Thione­(N_1_,N_9_) and one Thione­(N_1_,N_7_) is very similar in terms of band shape and relative Raman activity to the SERS spectrum experimentally measured. Therefore, we can hypothesize, for the first time, the presence on the gold nanoparticle of the two most populated tautomers of 6-MP.

### Influence of Adsorption Distance on Charge Transfer and Raman Response

To rationalize the origin of the observed Raman/SERS enhancements, we analyzed how the electronic structure and charge redistribution evolve along a controlled approach of 6-MP toward the Au_20_ cluster. As a representative case, the Thione­(N_1_,N_9_)@Au_20_ was selected to evaluate the total charge distribution over the two fragments—the Au_20_ cluster and the 6-MP molecule—while progressively increasing the N_1_–H···Au distance from its value at the optimized minimum up to ∼9.50 Å. In this analysis, two complementary charge partitioning schemes, namely Mulliken and Natural Bond Orbitals (NBO) approaches, have been employed to highlight robust trends rather than absolute values in providing a consistent description of the charge transfer evolution. This modeling strategy aims to elucidate how the approach and adsorption of 6-MP onto the gold cluster affects the electronic structure, and, in particular, how the concomitant charge transfer modulates the SERS spectrum and the stabilization and delocalization of the frontier orbital that governs the electronic coupling with the metal. Since the 6-MP HOMO acts as the main donor level in the CT process, any shift in energy provides a direct indicator of the evolving interaction strength. By correlating these variations with the Raman activities of specific vibrational modes—such as the N_1_–H stretching and out of plane—we aimed to establish a link between orbital alignment, charge redistribution across the gold interface, and the selective enhancement of vibrational signals observed in the experimental SERS spectrum. See Table 8 for details. Panel A of Figure 10 illustrates how the adsorption process of 6-MP on the Au_20_ cluster affects the energy of the frontier orbital (HOMO). This orbital is the principal donor orbital and contains the electronic density that can be transferred to the gold nanocluster. At largest separation (∼7.50–9.50 Å), the HOMO energy remains nearly constant with a relative energy value of 0.04 eV compared to the reference. As the 6-MP approaches the gold cluster, a progressive stabilization of the HOMO is observed. In particular between 5.50 and 4.50 Å, the energy decreases by 0.01 eV indicating the onset of a more pronounced electronic interaction. The most significant change occurs between 4.00 and 3.00 Å, where the HOMO energy shifts from 0.01 eV to −0.15 eV, corresponding to a stabilization of 0.16 eV over a distance interval of only 1 Å. At the shortest distance considered (2.50 Å), the HOMO reaches its minimum relative energy of −0.16 eV. This progressive stabilization reflects stronger electronic coupling at short distances and increasing charge transfer to the gold surface. Notably, the slope of the energy stabilization becomes significantly steeper below 4.00 Å, suggesting that the adsorption process involves a threshold region in which the frontier orbital begins to delocalize effectively over the molecule–metal interface and contribute to the charge-transfer mechanism. This process is visualized in Figure 11 where the HOMO isosurfaces are reported for each N_1_–H···Au distance considered. The Mulliken and NBO charge analysis reported in Table 8 provides complementary perspective on the evolution of charge transfer during adsorption. Both charge schemes show the same trend and highlight a charge separation between the 6-MP and the Au nanocluster as a function of the molecular distance. For example at the largest distance (9.50 Å), the charge separation on each fragment is negligible and begins to increase below 5.50 Å, reaching the maximum value at 2.50 Å where Mulliken estimate of the net positive charge on 6-MP is +0.88 and +0.17 for NBO). Panel B of Figure 10 illustrates the evolution of the net dipole moment as a function of N_1_–H···Au distance. The charge redistribution is accompanied by a pronounced increase in the dipole moment of the system which follows a nonmonotonic trend. At largest separation is computed around 9.78 D and reaches the maximum at ∼13.08 D at 2.50 Å. This large value supports the notion of a strong polarization across the adsorbate–substrate interface. Importantly, the direction of charge flow—from 6-MP to the metal cluster—is fully consistent with this dipole trend. The computed value of 13 D at the shortest distance provides further evidence of substantial electronic interaction and CT character in the adsorbed state. An additional key insight from this analysis concerns the vibrational response of the 6-MP molecule upon interaction with the Au metal nanocluster. In particular, we focused on two vibrational normal modes localized on the N_1_–H group that is directly involved in the interaction with Au (see Table 8 and Figure 10). From harmonic frequency calculations at constrained geometries we estimate that as the 6-MP approaches the minimum-energy distance (∼2.50 Å, *vertex* configuration), the Raman activity of the out-of-plane and stretching modes (Figure 10 panels C and D, respectively) undergo a notable increase. Also in this case, the behavior is not strictly linear, there is a distance threshold around 5.50 Å below which the effect increases significantly.

**Figure 10:**
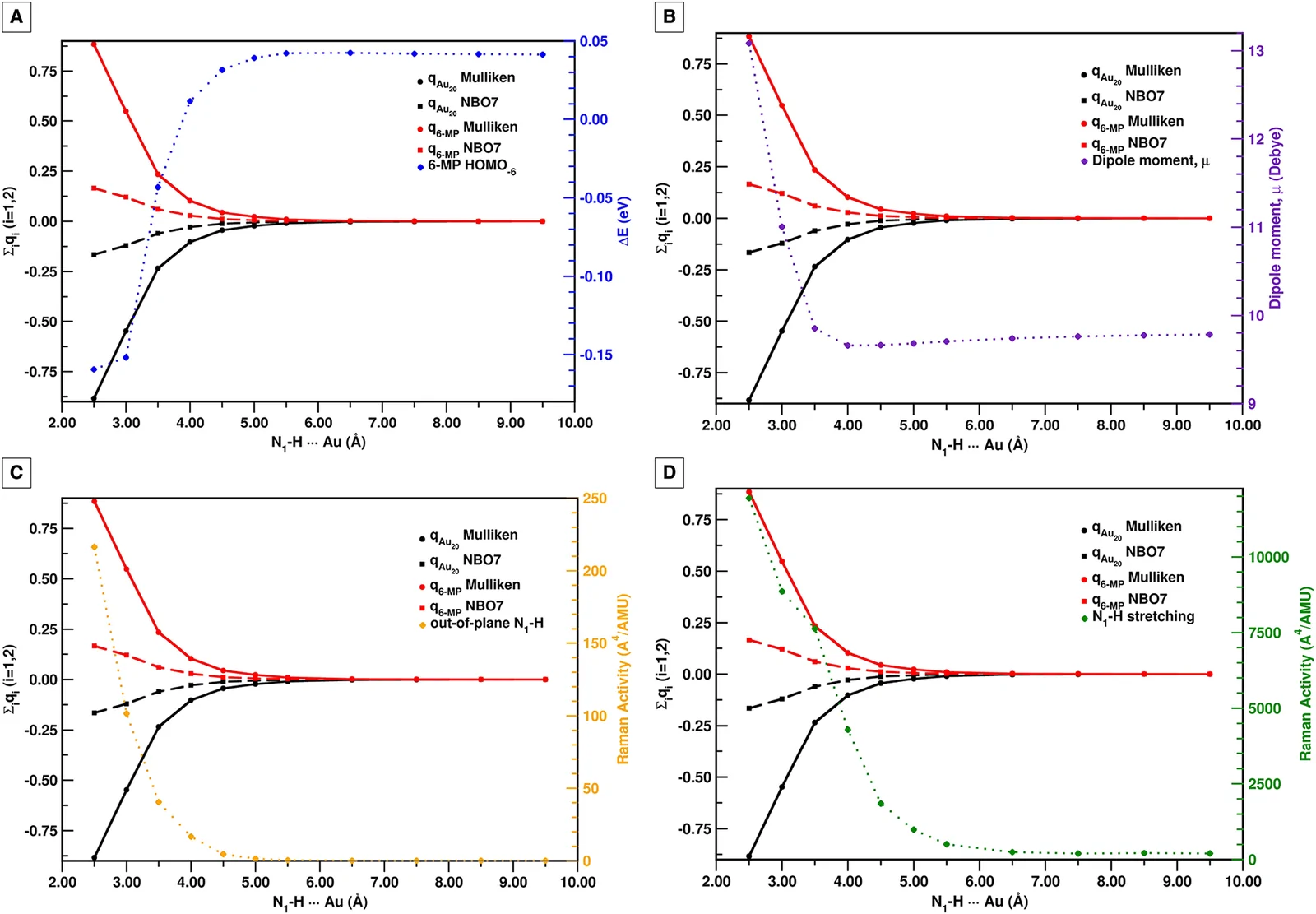
Panel A: Evolution of the total fragment charges and the energy of the 6-MP HOMO level relative to the HOMO of the isolated 6-MP as a function of the N_1_–H···Au distance. Panel B: Evolution of the total fragment charges and the dipole moment as a function of the N_1_–H···Au distance. Panel C: Evolution of the total fragment charges and the Raman activity of the N_1_–H out-of-plane bending mode as a function of the N_1_–H···Au distance. Panel D: Evolution of the total fragment charges and the Raman activity of the N_1_–H stretching mode as a function of the N_1_–H···Au distance. Mulliken charges (dots) and NBO charges (squares) are shown in black for the Au_20_ fragment and in red for 6-MP.

**Figure 11:**
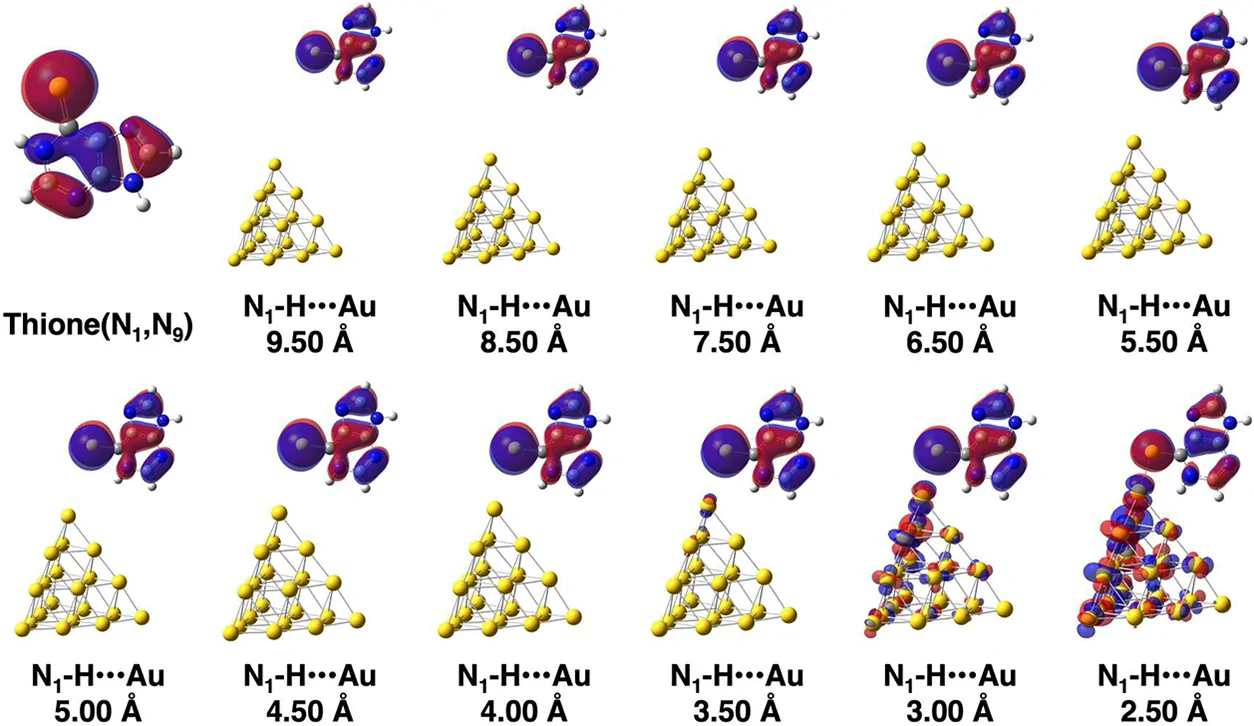
Contour plots (isovalue = 0.02) representation of the frontier orbital of Thione­(N_1_,N_9_) and the Thione­(N_1_,N_9_)@Au_20_ models at different N_1_–H···Au distances. The energy trend is reported in Table 8 and in Figure 10 Panel A.

**Table 8:** Computed Properties of the Thione­(N_1_,N_9_)@Au_20_ Complex as a Function of the N_1_–H···Au Distance (in Å)^a^

N_1_–H···Au	Mulliken ±*q*	NBO ±*q*	Dipole Moment μ	6-MP HOMO Δ*E*	Raman Activity out-of-plane N_1_–H	Raman Activity N_1_–H stretching
2.50	0.88	0.17	13.08	–0.16	216	11,939
3.00	0.55	0.12	11.00	–0.15	101	8864
3.50	0.23	0.06	9.85	–0.04	40	7631
4.00	0.10	0.03	9.66	0.01	17	4291
4.50	0.04	0.01	9.66	0.03	5	1845
5.00	0.02	0.01	9.68	0.04	1	989
5.50	0.00	0.00	9.70	0.04	0	509
6.50	0.00	0.00	9.74	0.04	0	246
7.50	0.00	0.00	9.76	0.04	0	200
8.50	0.00	0.00	9.77	0.04	0	214
9.50	0.00	0.00	9.78	0.04	0	202

a Reported values include the net charge on the 6-MP (+ sign) and Au fragments (− sign) estimated by Mulliken and Natural Bond Orbital (NBO) population analyses, the net dipole moment (μ, in Debye), the relative energy of the 6-MP HOMO orbital with respect to the HOMO of the isolated 6-MP (in eV), and the Raman activities of the out-of-plane N_1_–H deformation mode and the N_1_–H stretching mode (in Å^4^/amu).

In summary, bringing 6-MP into close contact with Au_20_ (below ∼4 Å) triggers a concerted response—HOMO stabilization, charge separation/polarization, and a marked rise of N_1_–H Raman activities—consistent with a short-range chemical enhancement origin of the observed spectral features.

## Conclusion

In this work, we combined Density Functional Theory (DFT) calculations with spontaneous Raman and Surface-Enhanced Raman Spectroscopy (SERS) measurements to establish a molecular-level interpretation of the vibrational response of 6-mercaptopurine (6-MP) in both aqueous solution and on gold nanostructures. The calculations show that the experimental Raman spectrum is dominated by the two thione tautomers, Thione­(N_1_,N_7_) and Thione­(N_1_,N_9_), whereas thiol forms are not required to explain the observed spectral pattern. At the same time, isolated-molecule models are insufficient to reproduce the experimental Raman response quantitatively. Much closer agreement is obtained with X-ray-derived dimeric models, demonstrating that supramolecular organization and local hydrogen-bonding topology play a decisive role in shaping the solid-state vibrational fingerprint. In particular, the dimer containing Thione­(N_1_,N_9_) provides the best match to experiment, with an average deviation of 1.1 ± 0.6%.

Building on this foundation, we extended our modeling to the SERS regime, simulating the adsorption of 6-MP tautomers on the three-dimensional Au_20_ nanocluster. By explicitly considering different surface coverage scenarios and tautomeric ratios, we were able to assign the main vibrational signatures observed experimentally and evaluate the influence of adsorption geometry on signal enhancement. In particular, we investigated several configurations involving up to four adsorbed 6-MP molecules arranged in different Thione­(N_1_,N_9_)/Thione­(N_1_,N_7_) tautomeric ratio anchored at distinct sites of the Au_20_ cluster. This spatial distribution reflects both site-specific binding preferences and cooperative interactions among adsorbates. The resulting simulated spectra closely match the experimental SERS profile, supporting the role of heterogeneous tautomeric populations and coverage-dependent effects in shaping the observed vibrational features. Our results show that both tautomer identity and intermolecular interactions contribute significantly to the SERS spectral response and must be carefully accounted in the modeling of drug-surface interactions. To our knowledge, this represents the first theoretical investigation to systematically explore multiple tautomeric forms of 6-MP on metal nanoparticle surface within a chemically consistent framework. More broadly, this work highlights a limitation of the conventional 1:1 molecule–cluster picture often used in computational SERS studies: for flexible, multitautomeric analytes such as 6-MP, the observed spectrum can arise from an ensemble of adsorbed species whose relative contributions depend on surface coverage, local packing, and binding site. This perspective should be useful for interpreting SERS spectra of other pharmacologically relevant molecules on gold nanostructures.

To gain additional insight into the origin of chemical enhancement, we performed a systematic analysis of how the interaction strength between 6-MP and the Au_20_ cluster affects frontier orbital energetics, charge redistribution, dipole moments, and Raman intensities. By progressively increasing the N_1_–H···Au distance, we correlated the stabilization of the HOMO and the onset of charge transfer with the selective amplification/enhancement of vibrational modes. This distance-dependent framework allowed us to identify a threshold region (∼4.5–5.0 Å) below which the electronic coupling and Raman activity sharply increase, establishing a direct link between orbital delocalization and vibrational enhancement.

Several promising avenues remain for future investigations. These include incorporation of anharmonic and thermal effects, the extension to larger or periodic metal models to better represent realistic experimental substrates, and the application of time-dependent DFT framework to explicitly compute resonant charge-transfer contributions to the SERS enhancement.

## Supplementary Material



## Notes

Comparison of experimental Raman and SERS spectra of 6-mercaptopurine; 6-MP tautomers in hybrid implicit/explicit solvation, including relative energies, Boltzmann populations, and reference Raman shifts/activities; crystal-derived dimers with atom labeling, X-ray vs optimized structural metrics, and Raman band assignments; simulated absorption spectra of 6-MP tautomers; optimized Au_20_ adsorption models (Thione­(N_1_,N_7_) and Thione­(N_1_,N_9_)) with simulated Raman spectra, including *flat* site, coverage effects (1–4 units), and mixed-tautomer stoichiometries (1:1, 2:1, 3:1, 2:2) compared to SERS; experimental peak tables and computed peak matching; normal-mode displacement vectors for a representative SERS-active complex (PDF)

## References

[1] Montgomery J. A. (1970). 2 The Biochemical
Basis for the Drug Actions of Purines. Prog.
Med. Chem..

[2] Sahasranaman S., Howard D., Roy S. (2008). Clinical pharmacology and pharmacogenetics
of thiopurines. Eur. J. Clin. Pharmacol..

[3] Nielsen O. H., Vainer B., Rask-Madsen J. (2001). the treatment
of inflammatory bowel
disease with 6-mercaptopurine or azathioprine. Aliment. Pharmacol. Ther..

[4] Lennard L. (1992). The clinical
pharmacology of 6-mercaptopurine. Eur. J. Clin.
Pharmacol..

[5] Relling M.
V., Hancock M. L., Boyett J. M., Pui C.-H., Evans W. E. (1999). Prognostic
importance of 6-mercaptopurine dose intensity in acute lymphoblastic
leukemia. Blood.

[6] Paugh S. W., Stocco G., Evans W. E. (2010). Pharmacogenomics
in pediatric leukemia. Curr. Opin. Pediatr..

[7] Larsen R. H., Hjalgrim L. L., Grell K., Kristensen K., Pedersen L. G., Brünner E. D., Als-Nielsen B., Schmiegelow K., Nersting J. (2020). Pharmacokinetics of
tablet and liquid
formulations of oral 6-mercaptopurine in children with acute lymphoblastic
leukemia. Cancer Chemother. Pharmacol..

[8] Andersen J. B., Szumlanski C., Weinshilboum R., Schmiegelow K. (1998). Pharmacokinetics,
dose adjustments, and 6-mercaptopurine/methotrexate drug interactions
in two patients with thiopurine methyltransferase deficiency. Acta Paediatr..

[9] Kang J.-S., Lee M.-H. (2009). Overview of therapeutic
drug monitoring. Korean J. Intern. Med..

[10] Alnaim L. (2007). Therapeutic
drug monitoring of cancer chemotherapy. J. Oncol.
Pharm. Pract..

[11] Supandi S., Harahap Y., Harmita H., Andalusia R. (2018). Simultaneous
analysis of 6-mercaptopurine, 6-methylmercaptopurine, and 6-thioguanosine-5′-monophosphate
in dried blood spot using ultra performance liquid chromatography
tandem mass spectrometry. Indones.J. Chem..

[12] Sato Y., Ishikawa Y., Matsuura H., Uosaki K., Mizutani F., Niwa O. (2005). Highly-Sensitive Determination
of 6-Mercaptopurine and Its Metabolites
by Electrochemical Reductive Desorption Measurements. Electroanalysis.

[13] Zhou P., He L., Gan G., Ni S., Li H., Li W. (2012). Fabrication
and Evaluation of [Co­(phen)_2_L]^3+^-Modified DNA–MWCNT
and SDS–MWCNT Electrodes for Electrochemical Detection of 6-Mercaptopurine. J. Electroanal. Chem..

[14] Keyvanfard M., Khosravi V., Karimi-Maleh H., Alizad K., Rezaei B. (2013). Voltammetric
determination of 6-mercaptopurine using a multiwall carbon nanotubes
paste electrode in the presence of isoprenaline as a mediator. J. Mol. Liq..

[15] Chrzanowska M., Sloderbach A. (1997). Spectrophotometric determination
of 6-mercaptopurine
and 6-thiouric acid by means of induced iodine-azide reaction. Chem. Anal..

[16] Diamai S., Warjri W., Saha D., Negi D. P. (2018). Sensitive determination
of 6-mercaptopurine based on the aggregation of phenylalanine-capped
gold nanoparticles. Colloids Surf., A.

[17] Jaworska A., Fornasaro S., Sergo V., Bonifacio A. (2016). Potential
of surface enhanced Raman spectroscopy (SERS) in therapeutic drug
monitoring (TDM). A critical review. Biosensors.

[18] Tommasini M., Zanchi C., Lucotti A., Bombelli A., Villa N. S., Casazza M., Ciusani E., de Grazia U., Santoro M., Fazio E., Neri F., Trusso S., Ossi P. M. (2019). Laser-synthesized SERS substrates
as sensors toward
therapeutic drug monitoring. Nanomaterials.

[19] Bonifacio A., Cervo S., Sergo V. (2015). Label-free
surface-enhanced Raman
spectroscopy of biofluids: fundamental aspects and diagnostic applications. Anal. Bioanal. Chem..

[20] Fleischmann M., Hendra P. J., McQuillan A. J. (1974). Raman spectra
of pyridine adsorbed
at a silver electrode. Chem. Phys. Lett..

[21] Jeanmaire D. L., Van Duyne R. P. (1977). Surface
Raman spectroelectrochemistry: Part I. Heterocyclic,
aromatic, and aliphatic amines adsorbed on the anodized silver electrode. J. Electroanal. Chem. Interfacial Electrochem..

[22] Albrecht M. G., Creighton J. A. (1977). Anomalously
intense Raman spectra of pyridine at a
silver electrode. J. Am. Chem. Soc..

[23] Langer J., Jimenez de Aberasturi D., Aizpurua J., Alvarez-Puebla R. A., Auguié B., Baumberg J. J., Bazan G. C., Bell S. E. J., Boisen A., Brolo A. G., Choo J., Cialla-May D., Deckert V., Fabris L., Faulds K., García
de Abajo F. J., Goodacre R., Graham D., Haes A. J., Haynes C. L., Huck C., Itoh T., Käll M., Kneipp J., Kotov N. A., Kuang H., Le Ru E. C., Lee H. K., Li J.-F., Ling X. Y., Maier S. A., Mayerhöfer T., Moskovits M., Murakoshi K., Nam J.-M., Nie S., Ozaki Y., Pastoriza-Santos I., Perez-Juste J., Popp J., Pucci A., Reich S., Ren B., Schatz G. C., Shegai T., Schlücker S., Tay L.-L., Thomas K. G., Tian Z.-Q., Van Duyne R. P., Vo-Dinh T., Wang Y., Willets K. A., Xu C., Xu H., Xu Y., Yamamoto Y. S., Zhao B., Liz-Marzán L. M. (2020). Present
and Future of Surface-Enhanced Raman Scattering. ACS Nano.

[24] Lombardi J. R., Birke R. L. (2009). A unified view of surface-enhanced Raman scattering. Acc. Chem. Res..

[25] Birke R. L., Lombardi J. R. (2015). Simulation of SERS by a DFT study: A comparison of
static and near-resonance Raman for 4-mercaptopyridine on small Ag
clusters. J. Opt..

[26] Tehrani, A. M. ; Mohaghegh, F. ; Materny, A. Raman Spectroscopy: Advances and Applications; Springer, 2024; pp 167–198.

[27] Han X. X., Rodriguez R. S., Haynes C. L., Ozaki Y., Zhao B. (2021). Surface-enhanced
Raman spectroscopy. Nat. Rev. Methods Primers.

[28] Majee, B. P. ; Mishra, A. K. Modern Techniques of Spectroscopy: Basics, Instrumentation, and Applications; Springer, 2021; pp 185–208.

[29] Le
Ru E. C., Blackie E., Meyer M., Etchegoin P. G. (2007). Surface
enhanced Raman scattering enhancement factors: a comprehensive study. J. Phys. Chem. C.

[30] Barbiellini B. (2017). Enhancement
of Raman scattering from molecules placed near metal nanoparticles. Low Temp. Phys..

[31] Ma H., Pan S.-Q., Wang W.-L., Yue X., Xi X.-H., Yan S., Wu D.-Y., Wang X., Liu G., Ren B. (2024). Surface-enhanced
Raman spectroscopy: current understanding, challenges, and opportunities. ACS Nano.

[32] Itoh T., Procházka M., Dong Z.-C., Ji W., Yamamoto Y. S., Zhang Y., Ozaki Y. (2023). Toward a new era of SERS and TERS
at the nanometer scale: From fundamentals to innovative applications. Chem. Rev..

[33] Morton S. M., Jensen L. (2009). Understanding the molecule- surface
chemical coupling
in SERS. J. Am. Chem. Soc..

[34] Lindquist N. C., de Albuquerque C. D. L., Sobral-Filho R. G., Paci I., Brolo A. G. (2019). High-speed
imaging of surface-enhanced Raman scattering fluctuations from individual
nanoparticles. Nat. Nanotechnol..

[35] Stefancu A., Aizpurua J., Alessandri I., Bald I., Baumberg J. J., Besteiro L. V., Christopher P., Correa-Duarte M., de Nijs B., Demetriadou A., Frontiera R. R., Fukushima T., Halas N. J., Jain P. K., Kim Z. H., Kurouski D., Lange H., Li J.-F., Liz-Marzán L. M., Lucas I. T., Meixner A. J., Murakoshi K., Nordlander P., Peveler W. J., Quesada-Cabrera R., Ringe E., Schatz G. C., Schlücker S., Schultz Z. D., Tan E. X., Tian Z.-Q., Wang L., Weckhuysen B. M., Xie W., Ling X. Y., Zhang J., Zhao Z., Zhou R.-Y., Cortés E. (2024). Impact of
Surface Enhanced Raman Spectroscopy in Catalysis. ACS Nano.

[36] Lian S., Li X., Lv X. (2025). Recent Developments in SERS Microfluidic Chips: From
Fundamentals to Biosensing Applications. ACS
Appl. Mater. Interfaces.

[37] Zhang Y., Mi X., Tan X., Xiang R. (2019). Recent progress on liquid biopsy
analysis using surface-enhanced Raman spectroscopy. Theranostics.

[38] Muniz-Miranda M., Muniz-Miranda F., Menziani M. C., Pedone A. (2023). Can DFT Calculations
Provide Useful Information for SERS Applications?. Molecules.

[39] Wang Q., Lian S., Guo C., Gao X., Dou Y., Song C., Lin J. (2022). The chemical adsorption effect of
surface enhanced Raman spectroscopy of nitrobenzene and aniline using
the density functional theory. Spectrochim.
Acta, Part A.

[40] Lian S., Li X., Lv X. (2024). Density functional theory study on the interaction
between aflatoxin B1/M1 and gold substrate. Langmuir.

[41] Zanchi C., Lucotti A., Pistaffa M., Ossi P. M., Trusso S., Fontana F., Carminati G., Rizzo S., Tommasini M. (2020). A Raman and
SERS study on the interactions of aza [5] helicene and aza [6] helicene
with a nanostructured gold surface. Vib. Spectrosc..

[42] Zanchi C., Tommasini M., Lucotti A., Pistaffa M., Trusso S., Ossi P., Fontana F., Bertolotti B., Rizzo S. (2024). SERS and DFT as Powerful
Tools in the Structure Elucidation of a
Novel Aza-Aromatic Derivative. J. Phys. Chem.
C.

[43] Chen R., Jensen L. (2020). Quantifying the enhancement mechanisms of surface-enhanced
Raman scattering using a Raman bond model. J.
Chem. Phys..

[44] Chaudhry I., Hu G., Ye H., Jensen L. (2024). Toward modeling the complexity of
the chemical mechanism in SERS. ACS Nano.

[45] Giovannini T., Gómez S., Cappelli C. (2025). Modeling raman spectra in complex
environments: From solutions to surface-enhanced raman scattering. J. Phys. Chem. Lett..

[46] Illobre P. G., Lafiosca P., Bonatti L., Giovannini T., Cappelli C. (2025). Mixed atomistic–implicit quantum/classical approach
to molecular nanoplasmonics. J. Chem. Phys..

[47] Zozulya A., Zyubin A., Samusev I. (2025). A review for DFT in chemical mechanism
of SERS studies. R. Soc. Open Sci..

[48] Truong D. H., Nguyen T. L. A., Ngo T. C., Dao D. Q. (2024). SERS-Based Sensor
Using Subnanometric Copper–Silver Mixed Clusters Ag (8–n)
Cu n (n= 0–8) for Pyridine: A DFT Study. J. Phys. Chem. A.

[49] Seuret-Hernández H. Y., Gamboa-Suaréz A., Morera-Boado C. (2022). A DFT study of the adsorption and
surface enhanced Raman spectroscopy of pyridine on Au20, Ag20, and
bimetallic Ag8Au12 clusters. J. Mol. Graphics
Modell..

[50] Valley N., Greeneltch N., Van Duyne R. P., Schatz G. C. (2013). A look at the origin
and magnitude of the chemical contribution to the enhancement mechanism
of surface-enhanced Raman spectroscopy (SERS): Theory and experiment. J. Phys. Chem. Lett..

[51] Ye H., Chaudhry I., Jensen L. (2025). Quantifying Electrostatic Contributions
to the Nonresonant Chemical SERS Enhancement. J. Phys. Chem. C.

[52] Zhao, Jensen L., Schatz G. C. (2006). Pyridine-
Ag20 cluster: a model system for studying surface-enhanced Raman scattering. J. Am. Chem. Soc..

[53] Jensen L., Zhao L. L., Schatz G. C. (2007). Size-dependence of the enhanced Raman
scattering of pyridine adsorbed on Ag n (n = 2 – 8, 20) Clusters. J. Phys. Chem. C.

[54] Viudez A. J., Madueno R., Pineda T., Blazquez M. (2006). Stabilization of gold
nanoparticles by 6-mercaptopurine monolayers. Effects of the solvent
properties. J. Phys. Chem. B.

[55] Pannico M., Musto P. (2021). SERS spectroscopy for
the therapeutic drug monitoring of the anticancer
drug 6-Mercaptopurine: Molecular and kinetic studies. Appl. Surf. Sci..

[56] Zheng J., Cheng X., Zhang H., Bai X., Ai R., Shao L., Wang J. (2021). Gold nanorods: the most versatile
plasmonic nanoparticles. Chem. Rev..

[57] Kumar S. S., Athimoolam S., Sridhar B. (2015). XRD, vibrational spectra and quantum
chemical studies of an anticancer drug: 6-Mercaptopurine. Spectrochim. Acta, Part A.

[58] Kersten K. M., Matzger A. J. (2016). Improved pharmacokinetics of mercaptopurine
afforded
by a thermally robust hemihydrate. Chem. Commun..

[59] Pazderski L., Łakomska I., Wojtczak A., Szłyk E., Sitkowski J., Kozerski L., Kamieński B., Koźmiński W., Tousek J., Marek R. (2006). The studies
of tautomerism in 6-mercaptopurine derivatives by 1H–13C, 1H–15N
NMR and 13C, 15N CPMAS-experimental and quantum chemical approach. J. Mol. Struct..

[60] Pannico M., Calarco A., Peluso G., Musto P. (2018). Functionalized gold
nanoparticles as biosensors for monitoring cellular uptake and localization
in normal and tumor prostatic cells. Biosensors.

[61] Pannico M., Musto P., Rega R., Vespini V., Grilli S., Ferraro P. (2020). Direct printing of
gold nanospheres from colloidal
solutions by pyro-electrohydrodynamic jet allows hypersensitive SERS
sensing. Appl. Surf. Sci..

[62] Becke A. D. (1993). Density-functional
thermochemistry. III. The role of exact exchange. J. Chem. Phys..

[63] Lee C., Yang W., Parr R. G. (1988). Development of the Colle-Salvetti
correlation-energy formula into a functional of the electron density. Phys. Rev. B.

[64] Vosko S. H., Wilk L., Nusair M. (1980). Accurate spin-dependent electron
liquid correlation energies for local spin density calculations: a
critical analysis. Can. J. Phys..

[65] Stephens P. J., Devlin F. J., Chabalowski C. F., Frisch M. J. (1994). Ab initio calculation
of vibrational absorption and circular dichroism spectra using density
functional force fields. J. Phys. Chem. A.

[66] Grimme S., Ehrlich S., Goerigk L. (2011). Effect of
the damping function in
dispersion corrected density functional theory. J. Comput. Chem..

[67] Frisch, M. J. ; Trucks, G. W. ; Schlegel, H. B. ; Scuseria, G. E. ; Robb, M. A. ; Cheeseman, J. R. ; Scalmani, G. ; Barone, V. ; Petersson, G. A. ; Nakatsuji, H. ; Li, X. ; Caricato, M. ; Marenich, A. V. ; Bloino, J. ; Janesko, B. G. ; Gomperts, R. ; Mennucci, B. ; Hratchian, H. P. ; Ortiz, J. V. ; Izmaylov, A. F. ; Sonnenberg, J. L. ; Williams-Young, D. ; Ding, F. ; Lipparini, F. ; Egidi, F. ; Goings, J. ; Peng, B. ; Petrone, A. ; Henderson, T. ; Ranasinghe, D. ; Zakrzewski, V. G. ; Gao, J. ; Rega, N. ; Zheng, G. ; Liang, W. ; Hada, M. ; Ehara, M. ; Toyota, K. ; Fukuda, R. ; Hasegawa, J. ; Ishida, M. ; Nakajima, T. ; Honda, Y. ; Kitao, O. ; Nakai, H. ; Vreven, T. ; Throssell, K. ; Montgomery, J. A., Jr. ; Peralta, J. E. ; Ogliaro, F. ; Bearpark, M. J. ; Heyd, J. J. ; Brothers, E. N. ; Kudin, K. N. ; Staroverov, V. N. ; Keith, T. A. ; Kobayashi, R. ; Normand, J. ; Raghavachari, K. ; Rendell, A. P. ; Burant, J. C. ; Iyengar, S. S. ; Tomasi, J. ; Cossi, M. ; Millam, J. M. ; Klene, M. ; Adamo, C. ; Cammi, R. ; Ochterski, J. W. ; Martin, R. L. ; Morokuma, K. ; Farkas, O. ; Foresman, J. B. ; Fox, D. J. Gaussian 16, Revision C.01; Gaussian Inc.: Wallingford CT, 2016.

[68] Barone V., Cossi M. (1998). Quantum calculation of molecular energies and energy gradients in
solution by a conductor solvent model. J. Phys.
Chem. A.

[69] Cossi M., Rega N., Scalmani G., Barone V. (2003). Energies, structures,
and electronic properties of molecules in solution with the C-PCM
solvation model. J. Comput. Chem..

[70] Groom C. R., Bruno I. J., Lightfoot M. P., Ward S. C. (2016). The Cambridge structural
database. Acta Crystallogr., Sect. B: Struct.
Sci., Cryst. Eng. Mater..

[71] Walker A. V. (2005). Structure
and energetics of small gold nanoclusters and their positive ions. J. Chem. Phys..

[72] Glendening E. D., Landis C. R., Weinhold F. (2019). NBO 7.0: New
vistas in localized
and delocalized chemical bonding theory. J.
Comput. Chem..

[73] O’boyle N. M., Tenderholt A. L., Langner K. M. (2008). Cclib: a library for package-independent
computational chemistry algorithms. J. Comput.
Chem..

[74] Polavarapu P. L. (1990). Ab initio
vibrational Raman and Raman optical activity spectra. J. Phys. Chem. A.

[75] Keresztury G., Holly S., Besenyei G., Varga J., Wang A., Durig J. (1993). Vibrational spectra of monothiocarbamates-II. IR and Raman spectra,
vibrational assignment, conformational analysis and ab initio calculations
of S-methyl-N, N-dimethylthiocarbamate. Spectrochim.
Acta, Part A.

[76] Meier, R. Handbook of Vibrational Spectroscopy; Chalmers, J. ; Griffiths, P. R. , Eds.; John Wiley & Sons Ltd.: Chichester, 2002; Vol. 1.

[77] Ivanov A. Y., Stepanian S., Karachevtsev V., Adamovicz L. (2022). Tautomers
of 6-thiopurine in low-temperature Ar matrices: FTIR spectroscopy
analysis and quantum mechanical calculations. Low Temper. Phys..

[78] Huyen D. T., Bui T. Q., Si N. T., Nhat P. V., Quy P. T., Nhung N. T. A. (2023). Theoretical study of the binding mechanism between
anticancerous drug mercaptopurine and gold nanoparticles using a cluster
model. J. Mol. Model..

[79] Pan X.-L., Cui F.-C., Liu J.-Y. (2011). Quantum mechanical/molecular
mechanical
molecular dynamics and free energy simulations of the Thiopurine S-Methyltransferase
reaction with 6-mercaptopurine. J. Phys. Chem.
B.

[80] Lapiński L., Nowak M. J., Kwiatkowski J. S., Leszczynski J. (1999). Phototautomeric
reaction, tautomerism, and infrared spectra of 6-thiopurine. Experimental
matrix isolation and quantum-mechanical (conventional ab initio and
density-functional theory) studies. J. Phys.
Chem. A.

[81] Gentile F.
S., Pannico M., Causa M., Mensitieri G., Di Palma G., Scherillo G., Musto P. (2020). Metal defects in HKUST-1
MOF revealed by vibrational spectroscopy: A combined quantum mechanical
and experimental study. J. Mater. Chem. A.

[82] Sousa S. F., Fernandes P. A., Ramos M. J. (2007). General performance of density functionals. J. Phys. Chem. A.

[83] Kryachko E. S., Remacle F. (2007). The magic gold cluster Au20. Int. J. Quantum Chem..

[84] Guo C., Song C., Wang Q., Dou Y., Gao X., Chen A., Lin J. (2023). Effects of Au6 and Au20 adsorption
sites of cyromazine–Au complexes by Raman spectroscopy and
density functional theory. Langmuir.

[85] Si N. T., Nhat P. V., Nguyen M. T. (2022). Binding
mechanism and SERS spectra
of 5-fluorouracil on gold clusters. Front. Chem..

[86] Mokkath J. H. (2023). Plasmon
induced hot carrier generation in a pyridine@ Au20 composite. Phys. Chem. Chem. Phys..

[87] Aikens C. M., Schatz G. C. (2006). TDDFT studies of absorption and SERS spectra of pyridine
interacting with Au20. J. Phys. Chem. A.

[88] Zhu M., Aikens C. M., Hollander F. J., Schatz G. C., Jin R. (2008). Correlating
the crystal structure of a thiol-protected Au25 cluster and optical
properties. J. Am. Chem. Soc..

[89] Lundberg L., Martini P., Goulart M., Gatchell M., Bohme D. K., Scheier P. (2019). Hydrogenated Gold clusters
from helium nanodroplets:
Cluster ionization and affinities for protons and hydrogen molecules. J. Am. Soc. Mass Spectrom..

[90] Gruene P., Rayner D. M., Redlich B., van der Meer A. F., Lyon J. T., Meijer G., Fielicke A. (2008). Structures
of neutral
Au7, Au19, and Au20 clusters in the gas phase. Science.

[91] Trang, N. V. ; Dao, D. Q. ; Nhat, P. V. ; Thuy, P. T. ; Nguyen, M. T. A Cluster model for interpretation of surface-enhanced raman scattering of organic compounds interacting with silver nanoparticles. In Practical Aspects of Computational Chemistry V; Springer, 2022; pp 255–285 10.1007/978-3-030-83244-5_8.

[92] Li J., Li X., Zhai H.-J., Wang L.-S. (2003). Au20: a tetrahedral
cluster. Science.

[93] Khudaberganov T. A., Khudobin P., Arakelian S. (2020). Magic Isomer
of a Au 20 Gold Nanocluster:
Topological Model and Spectral Characteristics. Bull. Russ. Acad. Sci.: Phys..

[94] Deriu C., Fabris L. (2025). A surface chemistry perspective on
SERS: revisiting
the basics to push the field forward. Chem.
Soc. Rev..

