# Supplementary material for: Molecular Modeling of the Raman and SERS Response of 6‑Mercaptopurine: Tautomerism, Surface Coverage, and Charge Transfer
Source: J Phys Chem C Nanomater Interfaces. 2026 Sep 27;130(40):13824–41. doi: 10.1021/acs.jpcc.6c03520 (PMC13660154; doi:10.1021/acs.jpcc.6c03520)
Supplement: Supplementary file 1 [file jp6c03520_si_001.pdf]

Electronic Supporting Information: Molecular Modeling of the Raman and SERS Response of 6-Mercaptopurine: Tautomerism, Surface Coverage, and Charge Transfer

Paola Cimino,[†] Pellegrino Musto,[‡] Marianna Pannico,[*,‡] Federico Coppola,[*,¶] and Nadia Rega[¶,§]

†*Department of Chemical Sciences, University of Napoli Federico II, Complesso Universitario di Monte S. Angelo, via Cintia 21, I-80126, Napoli, Italy*

‡*Institute for Polymers, Composites and Biomaterials, National Research Council of Italy, Via Campi Flegrei, 34, 80078 Pozzuoli, NA, Italy*

¶*Scuola Superiore Meridionale, Via Mezzocannone 4, I-80134, Napoli, Italy*

§*Istituto Nazionale Di Fisica Nucleare, sezione di Napoli, Complesso Universitario di Monte S. Angelo ed. 6, via Cintia 21, I-80126, Napoli, Italy*

E-mail: *marianna.pannico@cnr.it; *f.coppola@ssmeridionale.it

Contents

Figure S1: Comparison of experimental Raman and SERS spectra of 6-MP

Figure S2: 6-mercaptopurine tautomers modeled using a hybrid implicit/explicit solvation approach

Table S1: Boltzmann populations (%) at 298.15 K for all tautomeric pairs of 6-mercaptopurine, based on relative Gibbs free energies in C-PCM

Figure S3: Simulated absorption spectra of the 6-MP tautomers computed in hybrid implicit/explicit solvent model

Table S2: Raman shifts and corresponding Raman activities for various thione and thiol derivatives

Figure S4: Atom labeling scheme for the dimeric 6-mercaptopurine unit (deposition number 1447142)

Table S3: Comparison between computed and experimental bond lengths (deposition number 1447142)

Table S4: Comparison between computed and experimental bond angles (deposition number 1447142)

Table S5: Comparison between computed and experimental dihedral angles (deposition number 1447142)

Figure S5: Atom labeling scheme for the dimeric 6-mercaptopurine unit (deposition number 998661)

Table S6: Comparison between computed and experimental bond lengths (deposition number 998661)

Table S7: Comparison between computed and experimental bond angles (deposition number 998661)

Table S8: Comparison between computed and experimental dihedral angles (deposition number 998661)

Table S9: Comparison between observed and calculated Raman bands

Figure S6: Simulated Raman spectra of Thione(N_1,N_7) and Thione(N_1,N_9) tautomers adsorbed on Au_{20} in flat configuration

Figure S7: Optimized structures of Thione(N_1,N_9)@Au_{20} complexes containing one to four adsorbed 6-mercaptopurine units and corresponding normalized Raman spectra

Figure S8: Raman spectra of Thione(N_1,N_9)/Thione(N_1,N_7)@Au_{20} complexes in 1:1 ratio, compared with the SERS spectrum

Figure S9: Raman spectra of Thione(N_1,N_9)/Thione(N_1,N_7)@Au_{20} complexes in 2:1 ratio across different configurations

Figure S10: Structures of Thione(N_1,N_9)/(N_1,N_7)@Au_{20} complexes in a 3:1 ratio and corresponding Raman spectra compared to the experimental SERS spectrum

Figure S11: Raman spectra of Thione(N_1,N_9)/Thione(N_1,N_7)@Au_{20} complexes in 2:2 stoichiometry, including six distinct arrangements

Table S10: Experimental SERS peaks positions for 6-mercaptopurine

Figure S12: Normal modes displacement vectors of Thione(N_1,N_9)/Thione(N_1,N_7)@Au_{20} complex

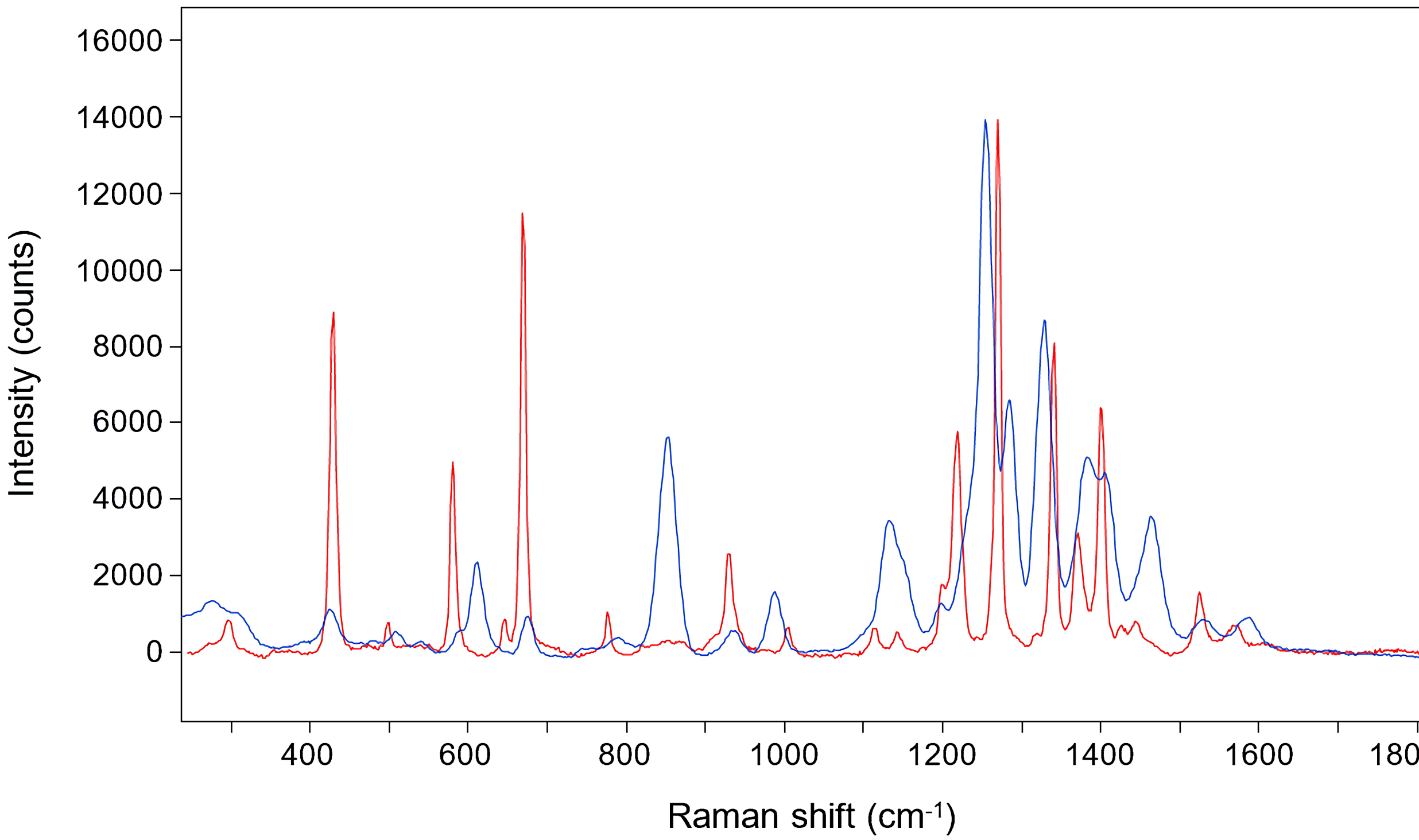

Figure S1. Comparison between the Raman (red) and the SERS (blue) spectra of 6-MP in the range 250 – 1800 cm^{-1}.

S0.1 Tautomeric forms and relative stability

The 6-mercaptopurine molecule can adopt several stable tautomeric forms, arising from the interplay of two distinct phenomena: (i) thione-thiol tautomerism, *i.e.*, the equilibrium between the thione form and the thiol (6-purinethiol, in *cis* or *trans* conformations) form, and (ii) prototropic tautomerism, involving the migration of the labile proton among the available nitrogen donor sites of the purine ring (N_1, N_3, N_7, N_9). In the following, we focus on the lowest-energy tautomers - four thione and two thiol isomers - which are expected to dominate under the investigated conditions. Table 1 presents the relative electronic energies (ΔE) and Gibbs free energies (ΔG) of each isomer compared to the most stable form in both gas phase and aqueous medium.

Consistently with previous studies,[1–4] the thione tautomers Thione(N_1,N_7) and Thione(N_1,N_9) emerge as the most stable configurations. It is important to emphasize that different tautomers are separated by only a few tenths of a kcal/mol, *i.e.*, within the intrinsic uncertainty of the compu-

tational protocol; they should therefore be considered quasi-degenerate and potentially coexisting depending on the local microenvironment.

In the gas phase, Thione(N_1,N_7) serves as the reference structure, whereas Thione(N_1,N_9) is slightly less stable, with an electronic energy difference of 3.09 kcal/mol. The thiol tautomers are significantly higher in energy, with Thiol(S_6,N_9) at ΔE= 3.82 kcal/mol and Thiol(S_6,N_7) at ΔE=7.70 kcal/mol. The Thione(N_3,N_7) tautomer is also notably less stable, with ΔE= 8.32 kcal/mol, indicating that shifting the tautomeric equilibrium towards this form is thermodynamically unfavorable. The less stable tautomer is Thione(N_3,N_9) with ΔE= 23.39 kcal/mol , making its formation highly unlikely under normal conditions. When solvation effects are introduced via the continuum solvation model, the stabilty ranking changes significantly. The relative energy between Thione(N_1,N_7) and Thione(N_1,N_9) decreases markedly to ΔE= 0.85 kcal/mol, suggesting a possible equilibrium between these two tautomers in aqueous solution. Meanwhile, the thiol forms remain substantially less stable, with Thiol(S_6,N_9) now at ΔE 7.67 kcal/mol and Thiol(S_6,N_7) at ΔE 9.86 kcal/mol. The Thione(N_3,N_7) is also stabilized by solvation, with ΔE 5.70 kcal/mol, although it remains significantly less favored compared to the N_1-substituted forms. The Thione(N_3,N_9) tautomer, while sligthly stabilized by solvation, remains the least stable, with an energy gap of more than 11 kcal/mol relative to reference species. To further refine our understanding of tautomer stability in aqueous environments, we extended our analysis by incorporating an explicit/implicit hybrid solvation approach, where four water molecules explicitly interact with each tautomer (Figure S2) while the bulk solvent is modeled using C-PCM. This cluster-continuum strategy allows us to better account for specific noncovalent interactions, such as hydrogen bonding, that are often poorly described by continuum models alone. This hybrid approach offers a more realistic representation of the chemical environment and provides a refined energetic and structural description of the tautomeric forms in solution. Notably, the Thione(N_1,N_9) tautomer becomes the most stable species in this model, while Thione(N_1,N_7) is now 0.13 kcal/mol. This inversion of stability underscores the significant role of explicit solute-solvent interactions in tautomeric equilibria. The thiol tautomers remain significantly less stable, with Thiol(S_6,N_9) at 7.72 kcal/mol and Thiol(S_6,N_7) at 10.23

kcal/mol. The Thione(N_3,N_7) is further stabilized by explicit solvation, reaching 2.70 kcal/mol, yet it remains higher in energy than the dominant N_1-substituted species. Finally, the Thione(N_3,N_9), despite stabilization, remains least favored, with ΔE 9.48 kcal/mol. The computed trend in Gibbs free energy largely follows those of the electronic energies, with minor variations due to entropic contributions. The thiol tautomers remain considerably less stable across all solvation models, reinforcing the dominance of thione forms in equilibrium conditions. Given these findings, in the following we will focus primarily on the Thione(N_1,N_9) and Thione(N_1,N_7) in C-PCM, examining their electronic and structural properties in greater detail. In this theoretical–computational work, we did not address the reaction pathways connecting the different tautomeric forms, nor did we compute the corresponding transition states or energy barriers. Our focus remains on the relative thermodynamic stabilities and spectroscopic features of the most populated 6-MP tautomers under equilibrium conditions. For all the possible tautomeric pairs, the relative Boltzmann populations have been also computed considering the Gibbs free energies obtained in C-PCM, all results are presented in Table S1.

Table S1. Relative Boltzmann populations (%) at 298.15 K for all possible tautomeric pairs in C-PCM. The energy difference, ΔG, is reported in millihartree (mHa), and the corresponding relative populations (P_1 and P_2) are computed using the Boltzmann distribution at room temperature. The Boltzmann constant used is $k_B = 3.1668 \times 10^{-3}$ mHa/K. The table is sorted in order of increasing energy difference ΔE.

**Tautomer$_1$**	**Tautomer$_2$**	**G_1 (Ha)**	**G_2 (Ha)**	**ΔG (mHa)**	**P_1 (%)**	**P_2 (%)**
Thione(N_1,N_7)	Thione(N_1,N_9)	-810.2711	-810.2697	1.40	81.4990	18.5010
Thiol(S_6,N_7)	Thione(N_3,N_9)	-810.2557	-810.2534	2.30	91.9530	8.0470
Thione(N_3,N_7)	Thiol(S_6,N_9)	-810.2621	-810.2591	3.00	95.9973	4.0027
Thiol(S_6,N_9)	Thiol(S_6,N_7)	-810.2591	-810.2557	3.40	97.3429	2.6571
Thiol(S_6,N_9)	Thione(N_3,N_9)	-810.2591	-810.2534	5.70	99.7617	0.2383
Thione(N_3,N_7)	Thiol(S_6,N_7)	-810.2621	-810.2557	6.40	99.8863	0.1137
Thione(N_1,N_9)	Thione(N_3,N_7)	-810.2697	-810.2621	7.60	99.9681	0.0319
Thione(N_3,N_7)	Thione(N_3,N_9)	-810.2621	-810.2534	8.70	99.9900	0.0100
Thione(N_1,N_7)	Thione(N_3,N_7)	-810.2711	-810.2621	9.00	99.9928	0.0072
Thione(N_1,N_9)	Thiol(S_6,N_9)	-810.2697	-810.2591	10.60	99.9987	0.0013
Thione(N_1,N_7)	Thiol(S_6,N_9)	-810.2711	-810.2591	12.00	99.9997	0.0003
Thione(N_1,N_9)	Thiol(S_6,N_7)	-810.2697	-810.2557	14.00	100.0000	0.0000
Thione(N_1,N_7)	Thiol(S_6,N_7)	-810.2711	-810.2557	15.40	100.0000	0.0000
Thione(N_1,N_9)	Thione(N_3,N_9)	-810.2697	-810.2534	16.30	100.0000	0.0000
Thione(N_1,N_7)	Thione(N_3,N_9)	-810.2711	-810.2534	17.70	100.0000	0.0000

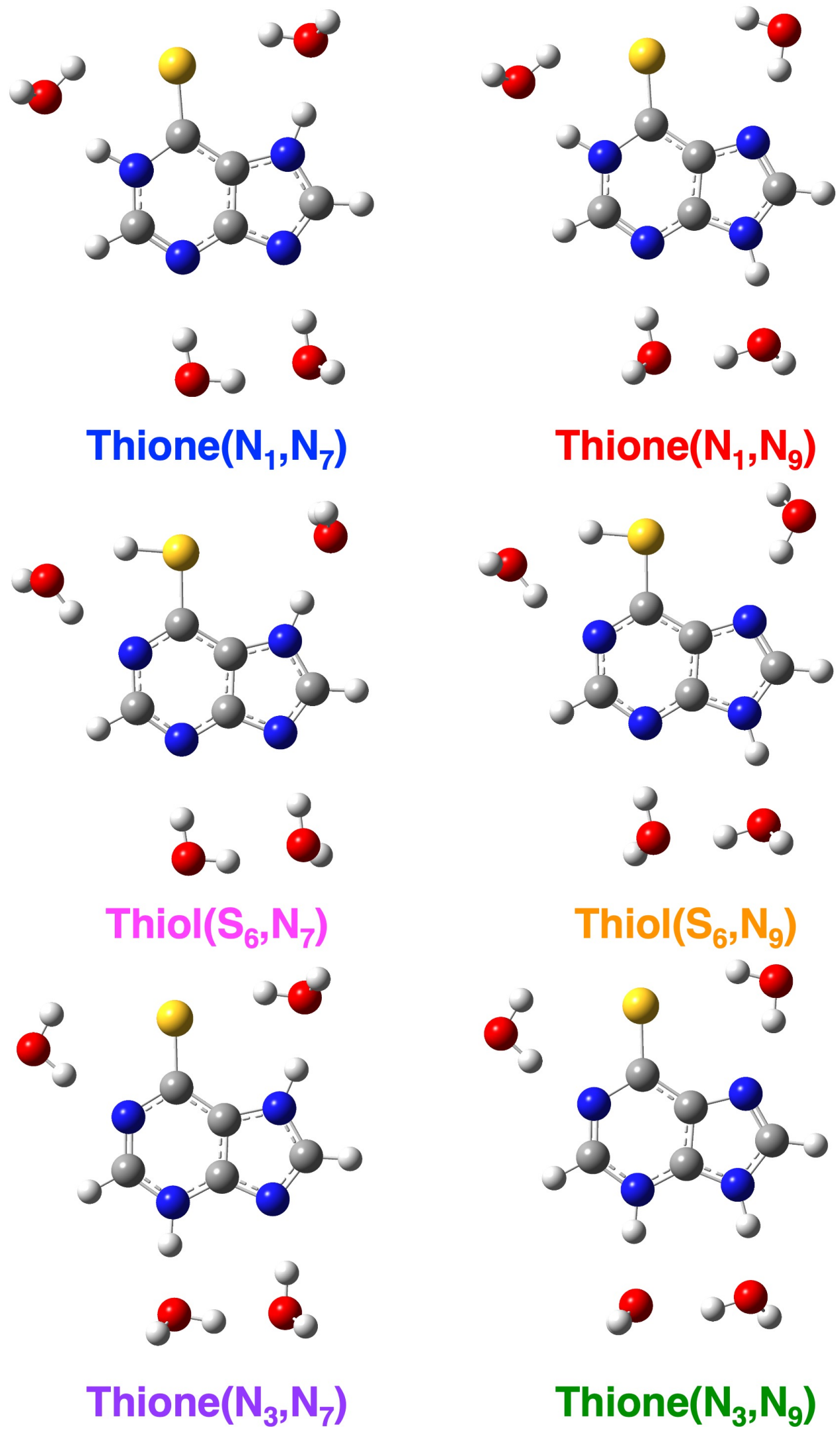

Figure S2. Optimized geometries of 6-mercaptopurine tautomers modeled using a hybrid implicit/explicit solvation approach, with four water molecules positioned at key hydrogen-bond donor and acceptor sites. Geometry optimizations were carried out at the B3LYP/6-311+G(d,p)/GD3BJ/C-PCM level of theory.

Table S2. Raman shifts (in cm^{-1}) and corresponding Raman activities (in $Å^4$/AMU) for various thione and thiol derivatives. The frequencies were computed at the DFT level using the B3LYP/6-311+G(d,p)/GD3BJ method, with the C-PCM (water) solvation model.

Thione(N_1,N_7)		Thione(N_1,N_9)		Thiol(S_6,N_7)		Thiol(S_6,N_9)		Thione(N_3,N_7)		Thione(N_3,N_9)	
**Raman Shift**	**Raman Activity**	**Raman Shift**	**Raman Activity**	**Raman Shift**	**Raman Activity**	**Raman Shift**	**Raman Activity**	**Raman Shift**	**Raman Activity**	**Raman Shift**	**Raman Activity**
125	0.2038	123	0.1653	124	1.6776	124	1.5268	111	0.4395	97	0.4874
198	0.2365	204	0.1253	191	3.2539	189	6.3353	190	0.1739	195	0.2042
200	15.5457	215	17.5360	211	3.8582	207	0.1003	204	15.7178	220	15.5044
265	1.1791	262	0.6715	247	2.0738	274	0.2339	280	0.1903	272	0.1035
416	31.1449	417	31.7645	286	0.1908	280	0.6194	419	29.8634	420	36.9219
490	3.7171	483	2.5087	388	5.8752	395	7.1521	468	3.2131	475	2.9152
527	2.3664	522	1.9980	481	0.2511	479	1.9832	494	1.0624	482	0.6445
548	0.5148	565	30.4253	486	3.9304	508	0.9472	555	1.0703	526	0.4008
570	30.0905	567	0.7358	528	1.7125	546	0.7121	565	26.9143	558	23.5732
580	0.7742	584	1.5838	567	14.5617	569	21.1243	585	0.0586	565	0.0764
645	0.8655	642	1.1059	601	0.0546	611	0.1876	630	1.1215	620	0.7112
656	46.4234	655	39.7967	640	1.0190	637	1.0083	652	67.3084	635	3.2694
685	0.1531	696	0.4420	660	37.8366	657	28.5115	654	0.9047	651	57.0840
754	1.7083	754	2.3288	767	0.6147	767	0.1865	737	1.9754	736	2.0239
839	9.0218	842	6.4525	826	40.0717	818	49.8777	847	5.2337	829	7.8205
861	4.6539	844	6.1460	872	2.1750	858	4.2334	853	6.0834	847	7.9430
916	1.4243	912	37.2037	886	19.8389	873	2.0888	923	0.6939	909	29.0585
924	33.9987	917	2.0503	916	3.8206	905	5.9404	924	27.5797	916	0.2730
981	26.3165	998	17.6804	931	0.8706	928	1.4990	970	12.8555	988	7.5465
1064	3.3990	1051	44.2932	971	63.4051	984	55.4643	1044	32.8623	1034	76.5082
1071	34.0355	1064	28.9005	1089	22.0959	1053	25.5063	1075	82.3521	1051	12.9930
1152	85.3671	1144	73.3839	1148	49.1093	1136	59.1387	1154	57.2680	1150	155.7846
1185	148.5142	1187	60.2842	1178	16.7915	1188	46.9767	1176	50.9499	1177	25.6200
1251	158.6063	1259	369.9506	1226	24.8112	1219	50.9719	1250	41.8829	1248	208.2615
1309	302.4250	1322	45.7368	1256	134.3339	1253	194.1951	1305	470.3185	1320	67.7276
1346	617.5615	1335	383.9210	1302	282.1458	1300	206.9786	1350	328.1288	1340	378.5215
1358	28.9325	1351	128.9717	1332	40.4935	1349	46.3616	1354	79.1630	1359	200.0682
1389	36.2316	1379	51.9569	1368	74.6985	1366	68.5374	1409	68.4084	1370	122.1561
1419	35.0174	1430	391.3696	1425	1.8469	1402	32.1572	1409	23.9523	1449	259.2481
1486	188.6224	1501	158.7803	1444	67.1498	1444	260.7325	1448	345.0188	1493	70.7070
1517	83.8174	1532	62.2004	1509	124.2491	1531	111.3741	1557	25.3149	1535	118.9029
1574	25.0344	1553	48.1116	1569	76.6023	1563	35.8314	1562	185.3875	1598	109.0440
3087	252.7518	3092	240.1181	2588	253.2987	2574	355.1393	3079	287.9987	3076	291.3292
3133	212.8782	3131	224.4212	3052	294.1666	3057	289.5428	3138	216.5397	3134	230.6882
3433	163.4338	3428	163.9932	3127	216.1637	3127	222.0851	3456	321.6127	3455	376.1766
3480	207.4525	3485	424.0188	3494	221.1238	3491	337.5721	3478	167.6024	3489	403.5980

S0.2 Optical properties: UV-Vis signatures of thione vs thiol tautomers

We performed a detailed analysis of the electronic absorption spectrum of 6-MP comparing the experimental UV-Vis spectra acquired in aqueous solution with the simulated absorption profiles of all six possible tautomers, modeled using a hybrid explicit/implicit solvation scheme (see Figure S2), in order to further characterize the 6-MP species mainly present in solution. The experimental spectrum (solid and dashed black lines in Figure S3) shows a broad band in the near-UV spanning 290-350 nm, with a maximum around 320-325 nm and a low energy tail beyond 350 nm. A mid-intense band is also observed in the mid-UV at 230 nm. The simulated spectra of the thione tautomers reproduce remarkably well the position of the low-energy absorption band which spans from 290-360 nm, with intense HOMO-LUMO transitions ($f \approx 0.4$-0.5) assigned to π-π^* excitations involving frontier orbitals localized on the purine ring. This excellent energetic and intensity correspondence supports a predominant thione contribution to the near-UV experimental band. In contrast, the thiol forms (orange and magenta) exhibit intense electronic transitions around 260-280 nm where the experimental optical spectra show only a weak and diffuse tail, which is consistent with weak thione transitions ($f \leq 0.1$), possibly complemented by a minor thiol contribution. The higher energies bands ($\lambda \leq 200$ nm) are less diagnostic, indeed appears that all tautomers contribute to the spectral congestion. To summarize, the good agreement between simulated and experimental absorption spectra, especially in the diagnostic low-energy region provides a further evidence that the species predominantly present in solution under experimental conditions are thione tautomers. The absence of characteristic thiol signatures supports the conclusion that thiol forms are either absent or present only in very negligible concentrations.

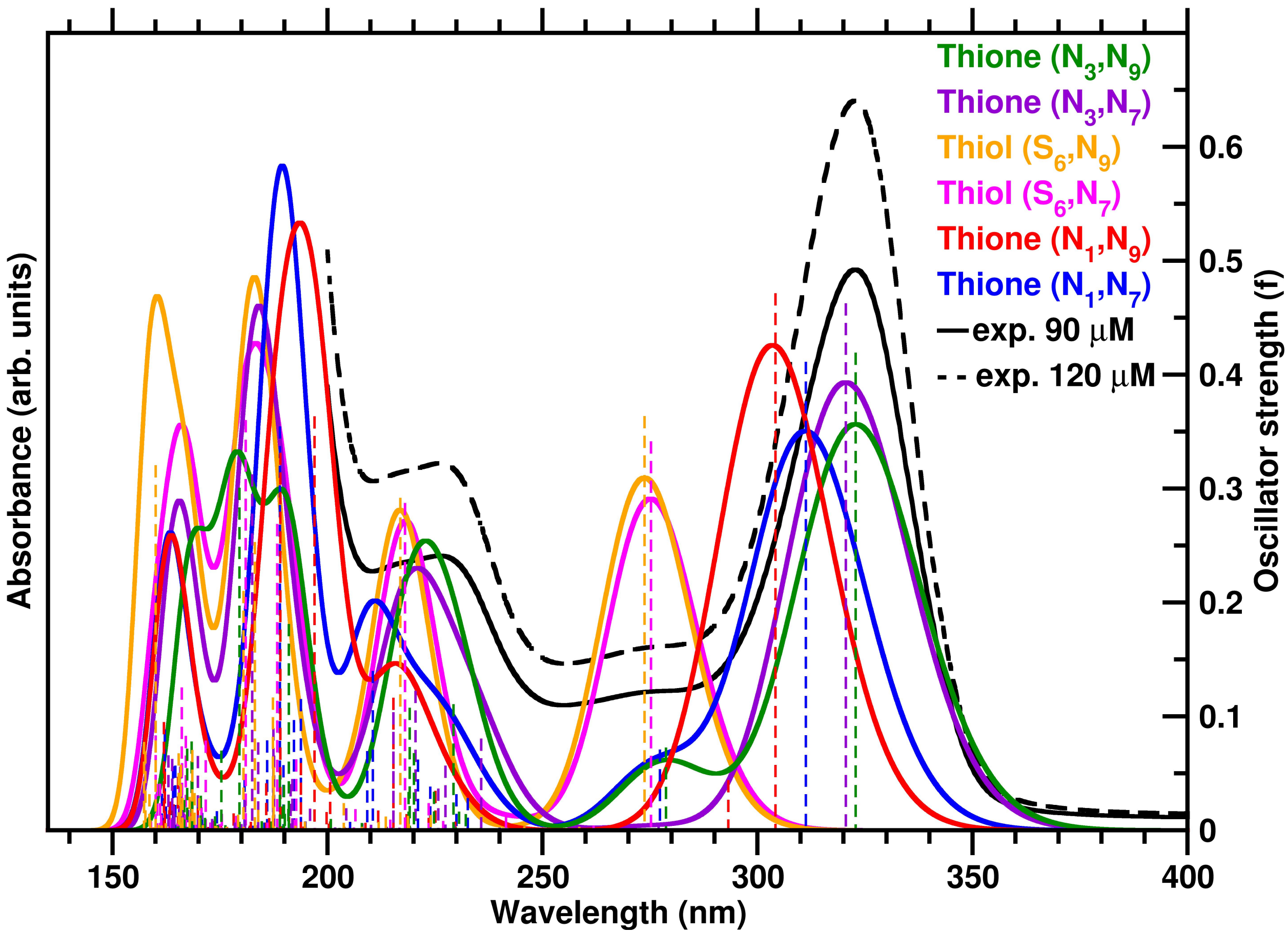

Figure S3. Simulated absorption spectra of the 6-MP tautomers computed in hybrid implicit/explicit solvent model, reported in Figure S2. A HWHM of 0.20 eV was employed for the gaussian convolution of the vertical excitation energies, reported as vertical dashed sticks. The experimental UV–Vis spectra recorded for 90 and 120 μM solutions are reported as solid and dashed black lines, respectively.

S0.3 Structural analysis of the X-ray-based dimer

A comparative evaluation of key structural parameters - such as bond lengths, bond angles, and dihedral torsions - was carried out between the optimized and experimental structure (see Figure S4 and Figure S5). Overall, bond lengths and angles in the optimized structure show excellent agreement with experimental values, with deviations within 0.02–0.03 Å and 1–3° for most covalent bonds and angles, respectively (see Tables S3 and S4 and Tables S6 and S7). Larger differences are found for X–H distances (especially N–H and O–H), which are typically underestimated in X-ray structures due to their low electron density and are often refined or constrained during crystallographic analysis. Dihedral angles also display a consistent pattern between the crystal and the optimized structure, with most torsional values deviating by less than 3–5°, indicating that the planarity and hydrogen-bonding topology are preserved (Tables S5 and S8). These observations validate the optimized dimer as a structurally reliable model of the crystalline state, suitable for subsequent vibrational and spectroscopic analysis.

S0.3.1 XRD analysis CSD, deposition number 1447142

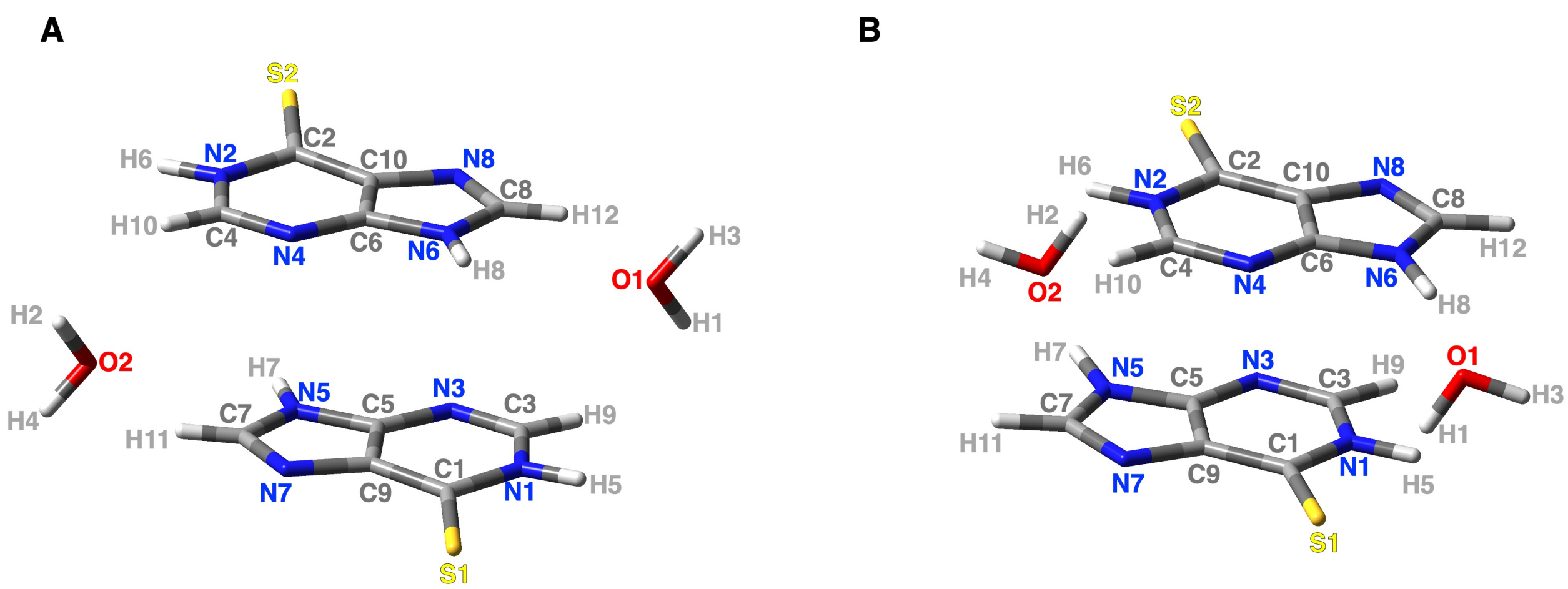

Figure S4. Atom labeling scheme for the dimeric 6-mercaptopurine unit as extracted from the experimental X-ray crystal structure (A) and after optimization at the B3LYP/6-311+G(d,p)/GD3BJ/C-PCM(water) level of theory (B).

Table S3. Computed bond lengths (in Å) for selected atom pairs in 6-mercaptopurine, optimized at the B3LYP/6-311+G(d,p)/GD3BJ/C-PCM level of theory. Values in parentheses refer to the experimental bond lengths measured from the X-ray crystal structure (CSD, deposition number 1447142). See Figure S4 for labeling scheme.

**Atom Pair**	**Length (Å)**
S1-C1	1.689 (1.676)
C1-N1	1.387 (1.393)
C1-C9	1.413 (1.406)
H5-N1	1.014 (0.993)
N1-C3	1.363 (1.353)
N7-C9	1.378 (1.372)
N7-C7	1.316 (1.316)
C9-C5	1.407 (1.398)
C3-H9	1.082 (0.931)
C3-N3	1.304 (1.318)
C7-H11	1.080 (0.930)
C7-N5	1.373 (1.367)
C5-N3	1.351 (1.352)
C5-N5	1.362 (1.360)
N5-H7	1.033 (0.856)
H1-O1	0.978 (0.961)
H4-O2	0.964 (0.968)
O1-H3	0.964 (0.968)
O2-H2	0.978 (0.961)
H8-N6	1.033 (0.856)
N6-C6	1.362 (1.360)
N6-C8	1.373 (1.367)
N4-C6	1.351 (1.352)
N4-C4	1.304 (1.318)
H12-C8	1.080 (0.930)
C6-C10	1.407 (1.398)
H10-C4	1.082 (0.931)
C8-N8	1.316 (1.316)
C4-N2	1.363 (1.353)
C10-N8	1.378 (1.372)
C10-C2	1.413 (1.406)
N2-H6	1.014 (0.993)
N2-C2	1.387 (1.393)
C2-S2	1.689 (1.676)

Table S4. Computed bond angles (in degrees, °) for selected atoms in 6-mercaptopurine, optimized at the B3LYP/6-311+G(d,p)/GD3BJ/C-PCM level of theory. Values in parentheses refer to the experimental bond angles measured from the X-ray crystal structure (CSD, deposition number 1447142). See Figure S4 for labeling scheme.

Bond angles	Angle (°)
H1-O1-H3	105.54 (100.32)
H2-O2-H4	105.54 (100.32)
H5-N1-C1	116.41 (117.10)
C1-N1-C3	125.76 (124.94)
H5-N1-C3	117.82 (117.94)
H6-N2-C4	117.82 (117.94)
C2-N2-C4	125.76 (124.94)
H6-N2-C2	116.41 (117.10)
C3-N3-C5	113.02 (112.06)
C4-N4-C6	113.02 (112.06)
C5-N5-C7	106.46 (106.72)
H7-N5-C7	127.06 (127.23)
H7-N5-C5	125.85 (125.87)
H8-N6-C6	125.86 (125.87)
H8-N6-C8	127.06 (127.23)
C6-N6-C8	106.46 (106.72)
C7-N7-C9	104.60 (104.57)
C8-N8-C10	104.60 (104.57)
S1-C1-N1	121.01 (121.46)
S1-C1-C9	127.71 (126.92)
N1-C1-C9	111.28 (111.61)
N2-C2-C10	111.28 (111.61)
S2-C2-C10	127.71 (126.92)
S2-C2-N2	121.01 (121.46)
N1-C3-H9	115.57 (117.59)
N1-C3-N3	124.11 (124.76)
N3-C3-H9	120.32 (117.65)
N4-C4-H10	120.32 (117.65)
N2-C4-N4	124.11 (124.76)
N2-C4-H10	115.57 (117.59)
N3-C5-C9	126.83 (127.27)
N5-C5-C9	105.71 (105.47)
N3-C5-N5	127.46 (127.26)
N4-C6-N6	127.46 (127.26)
N6-C6-C10	105.71 (105.47)
N4-C6-C10	126.83 (127.27)
N7-C7-H11	125.27 (123.50)
N5-C7-N7	113.26 (112.94)
N5-C7-H11	121.46 (123.56)
N6-C8-H12	121.46 (123.56)
N6-C8-N8	113.26 (112.94)
N8-C8-H12	125.27 (123.50)
N7-C9-C1	131.01 (130.68)
C1-C9-C5	119.00 (119.00)
N7-C9-C5	109.95 (110.29)
N8-C10-C6	109.95 (110.29)
C2-C10-C6	119.00 (119.00)
N8-C10-C2	131.01 (130.68)

Table S5. Computed dihedral angles (in degrees, °) for selected atoms in 6-mercaptopurine, optimized at the B3LYP/6-311+G(d,p)/GD3BJ/C-PCM level of theory. Values in parentheses refer to the experimental dihedral angles measured from the X-ray crystal structure (CSD, deposition number 1447142). See Figure S4 for labeling scheme.

**Dihedral**	**Torsion (°)**
N4–C6–C10–N8	-179.25 (-178.73)
N6–C6–C10–C2	178.23 (-177.33)
N8–C10–C2–N2	178.39 (175.92)
C4–N4–C6–N6	-178.66 (-177.98)
S2–C2–C10–C6	-178.66 (172.07)
N7–C9–C5–N3	174.25 (178.73)
C1–C9–C5–N5	-178.22 (177.33)
N1–C1–C9–N7	-178.39 (-175.92)
N5–C5–N3–C3	178.65 (177.98)
S1–C1–C9–C5	178.66 (-172.07)

S0.3.2 XRD analysis CSD, deposition number 998661

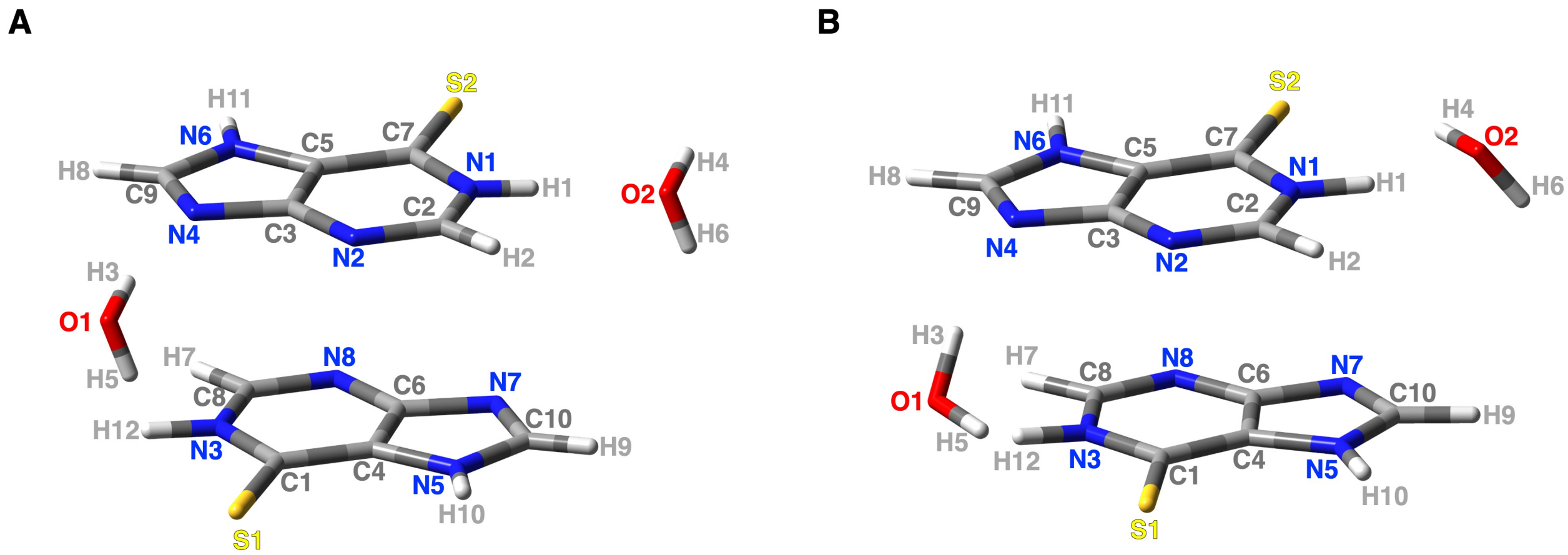

Figure S5. Atom labeling scheme for the dimeric 6-mercaptopurine unit as extracted from the experimental X-ray crystal structure (A) and after optimization at the B3LYP/6-311+G(d,p)/GD3BJ/C-PCM(water) level of theory (B).

Table S6. Computed bond lengths (in Å) for selected atom pairs in 6-mercaptopurine, optimized at the B3LYP/6-311+G(d,p)/GD3BJ/C-PCM level of theory. Values in parentheses refer to the experimental bond lengths measured from the X-ray crystal structure (CSD, deposition number 998661). See Figure S5 for labeling scheme.

**Atom Pair**	**Length (Å)**
H2-C2	1.083 (0.940)
H10-N5	1.011 (0.853)
S1-C1	1.687 (1.678)
C2-N2	1.305 (1.308)
C2-N1	1.363 (1.353)
N2-C3	1.357 (1.368)
N5-C10	1.362 (1.350)
N5-C4	1.372 (1.373)
H9-C10	1.079 (0.916)
H1-N1	1.031 (0.837)
H6-O2	0.964 (0.824)
O2-H4	0.975 (0.853)
H5-O1	0.970 (0.824)
N1-C7	1.383 (1.379)
C10-N7	1.323 (1.328)
C1-C4	1.406 (1.408)
C1-N3	1.383 (1.379)
C4-C6	1.404 (1.396)
C3-N4	1.373 (1.370)
C3-C5	1.401 (1.396)
O1-H3	0.979 (0.853)
N4-C9	1.326 (1.328)
N7-C6	1.370 (1.370)
C7-C5	1.407 (1.408)
C7-S2	1.688 (1.678)
N3-H12	1.028 (1.000)
N3-C8	1.363 (1.353)
C6-N8	1.359 (1.368)
C5-N6	1.373 (1.373)
C9-N6	1.358 (1.350)
C9-H8	1.078 (0.916)
C8-N8	1.304 (1.308)
C8-H7	1.083 (0.940)
N6-H11	1.011 (0.853)

Table S7. Computed bond angles (in degrees, °) for selected atoms in 6-mercaptopurine, optimized at the B3LYP/6-311+G(d,p)/GD3BJ/C-PCM level of theory. Values in parentheses refer to the experimental bond angles measured from the X-ray crystal structure (CSD, deposition number 998661). See Figure S5 for labeling scheme.

Bond angles	Angle (°)
C4–C1–S1	126.73 (126.99)
S1–C1–N3	122.91 (122.56)
C4–C1–N3	110.36 (110.44)
N2–C2–H2	119.91 (120.32)
N1–C2–H2	115.01 (114.28)
N1–C2–N2	125.07 (125.37)
N2–C3–N4	126.42 (125.76)
N2–C3–C5	123.59 (124.04)
C5–C3–N4	109.98 (110.20)
C1–C4–N5	132.38 (132.39)
N5–C4–C6	105.24 (105.70)
C1–C4–C6	122.38 (121.90)
C2–N1–H1	118.59 (115.75)
C2–N1–C7	124.75 (125.23)
H1–N1–C7	116.65 (119.00)
C2–N2–C3	113.88 (112.98)
H3–O1–H5	104.92 (107.08)
H4–O2–H6	105.80 (107.08)
C1–N3–H12	116.36 (117.38)
C1–N3–C8	124.92 (125.23)
C8–N3–H12	118.43 (117.38)
C3–C5–C7	122.19 (121.90)
C3–C5–N6	105.36 (105.70)
C7–C5–N6	132.44 (132.39)
C3–N4–C9	104.99 (104.24)
C10–N5–H10	126.96 (126.83)
C4–N5–H10	126.28 (126.81)
C4–N5–C10	106.76 (106.30)
C4–C6–N7	110.26 (110.20)
C4–C6–N8	123.35 (124.04)
N7–C6–N8	126.38 (125.76)
N1–C7–C5	110.49 (110.44)
N1–C7–S2	123.33 (122.56)
C5–C7–S2	126.18 (126.99)
N3–C8–N8	125.03 (125.37)
N3–C8–H7	115.00 (114.28)
N8–C8–H7	119.97 (120.32)
C5–N6–C9	106.99 (106.30)
C5–N6–H11	126.27 (126.81)
C9–N6–H11	126.73 (126.89)
N4–C9–N6	112.67 (113.57)
N4–C9–H8	125.28 (123.59)
N6–C9–H8	122.04 (122.84)
N5–C10–H9	121.67 (122.84)
N5–C10–N7	112.96 (113.57)
N7–C10–H9	125.37 (123.59)
C6–N7–C10	104.78 (104.24)
C6–N8–C8	113.94 (112.98)

Table S8. Computed dihedral angles (in degrees, °) for selected atoms in 6-mercaptopurine, optimized at the B3LYP/6-311+G(d,p)/GD3BJ/C-PCM level of theory. Values in parentheses refer to the experimental dihedral angles measured from the X-ray crystal structure (CSD, deposition number 998661). See Figure S5 for labeling scheme.

**Dihedral**	**Torsion (°)**
N2–C3–C5–N6	-179.95 (179.31)
N4–C3–C5–C7	-178.86 (-179.13)
N6–C5–C7–N1	-178.47 (-177.81)
C2–N2–C3–N4	178.34 (178.62)
S2–C7–C5–C3	-179.58 (-178.26)
N7–C6–C4–C1	179.62 (179.13)
N5–C4–C1–N3	178.33 (177.81)
N8–C6–C4–N5	-179.55 (-179.31)
C8–N8–C6–N7	-178.72 (-178.62)
S1–C1–C4–C6	179.39 (178.26)

Table S9. Comparison between observed and calculated Raman bands (Figure 4, right side), with relative normalized intensities in parentheses, percentage error, and vibrational assignments. pr = purine structure; ring 1 (r1) and ring 2 (r2) are identified in Figure 1, left side; ν = stretching; δ = bending; ω = wagging; the notation of wagging modes is: Atoms 1 and 3 define the ends of the four-atom fragment. Atom 2 is the central atom, and atom 4 is the substituent bonded to atom 2. The wagging mode corresponds to an out-of-plane motion of atom 4 relative to the plane formed by atoms 1–2–3.

Obs. (cm^{-1})	Calc. (cm^{-1})	Err. (%)	Description
296 (0.06)	277 (0.22)	6.4	Out-of-plane def pr
–	333 (0.09)		$w(H_2O)$
427 (0.64)	419 (0.79)	1.9	In-plane def r1
–	472 (0.38)		$w(H_2O)$
497 (0.06)	502 (0.18)	1.0	In-plane def pr/$w(H_2O)$
540 (0.02)	541 (0.35)	0.2	$w(H_2O)$
579 (0.36)	572 (0.50)	1.2	In-plane def pr
645 (0.06)	638 (0.08)	1.1	$w(H_2O)$/w(CNCH)r2
668 (0.82)	659 (0.59)	1.3	In-plane def pr
775 (0.08)	762 (0.03)	1.7	Out-of-plane def pr
912 (sh, 0.02)	–		
–	839 (0.07)		In-plane def pr/w(CNCH)r1
928 (0.19)	928 (0.18)	0.0	In-plane def r2/w(NCNH)r1/w(CNCH)r1
1003 (0.05)	981 (0.13)	2.2	In-plane def pr/δ(NCH)r2/δ(CNH)r2/ν(CS)
1114 (0.05)	1090 (0.13)	2.2	ν(CN)r1/ν(NC)r1/δ(CNH)r2
1142 (0.04)	–		
1198 (0.13)	1172 (0.17)	2.2	In-plane def pr/δ(NCH)r2/δ(CNH)r1/ν(CS)
1218 (0.42)	1193 (0.66)	2.1	ν(CN)ring1/ν(NC)ring1/δ(CNH)ring2/δ(NCH)ring2
1269 (1)	1252 (0.41)	1.3	In-plane def of pr/δ(NCH)r2
1317 (0.04)	–		
1340 (0.58)	1308 (0.83)	2.4	ν(CN)r2/δ(CCN)r1/δ(NCH)r1
1370 (0.23)	1360 (1)	0.73	In plane def pr/δ(CNH)r2/δ(NCH)r1
1377 (0.15)	–		
1400 (0.46)	1405 (0.17)	0.36	δ(CNH)r2/δ(NCH)r2/δ(CNH)r1/δ(NCH)r1/In plane def r2
1426 (0.05)	1432 (0.08)	0.42	In plane def r2/δ(CNH)r2/δ(NCH)r2
1443 (0.06)	–		
1525 (0.12)	1494 (0.25)	2.0	ν(CN)r1/in plane def r2/δ(NCH)r2/δ(NCH)r1
1539 (0.02)	–		
1558 (0.04)	1554 (0.08)	0.26	δ(CNH)r1/δ(NCH)r1/ν(CN)r1
1570 (0.05)	1567 (0.07)	0.19	δ(CNH)r1/δ(NCH)r1/ν(CN)r1/$\delta(H_2O)$
	$\overline{\text{Err}}$ = 1.2 ± 0.9		

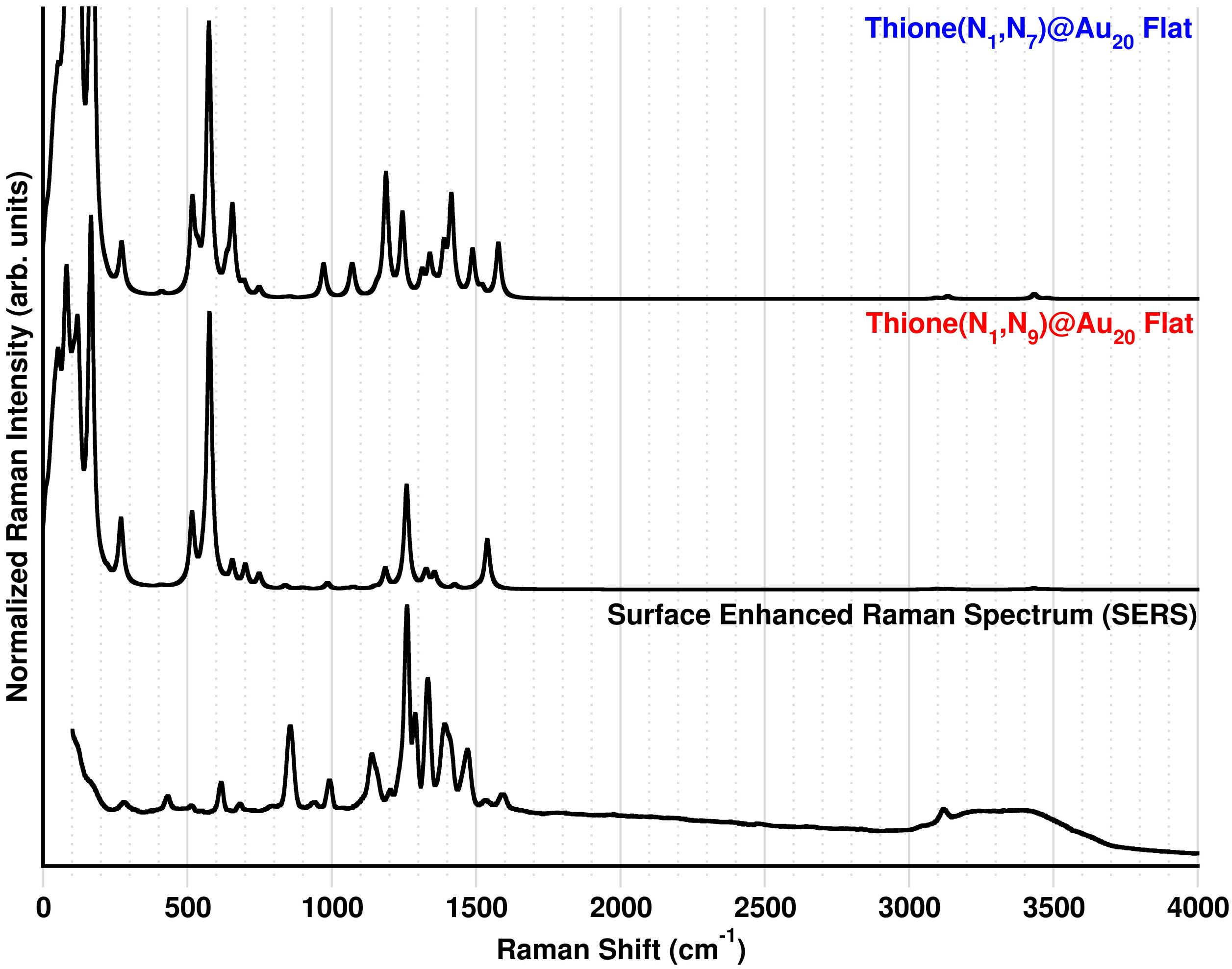

Figure S6. Normalized Raman spectra of Thione(N_1,N_7) and Thione(N_1,N_9) tautomers adsorbed on Au_{20} cluster in *flat* configuration computed at B3LYP/6-311+G(d,p)/GD3BJ/C-PCM level of theory. The experimental SERS spectrum is reported at the bottom for comparison.

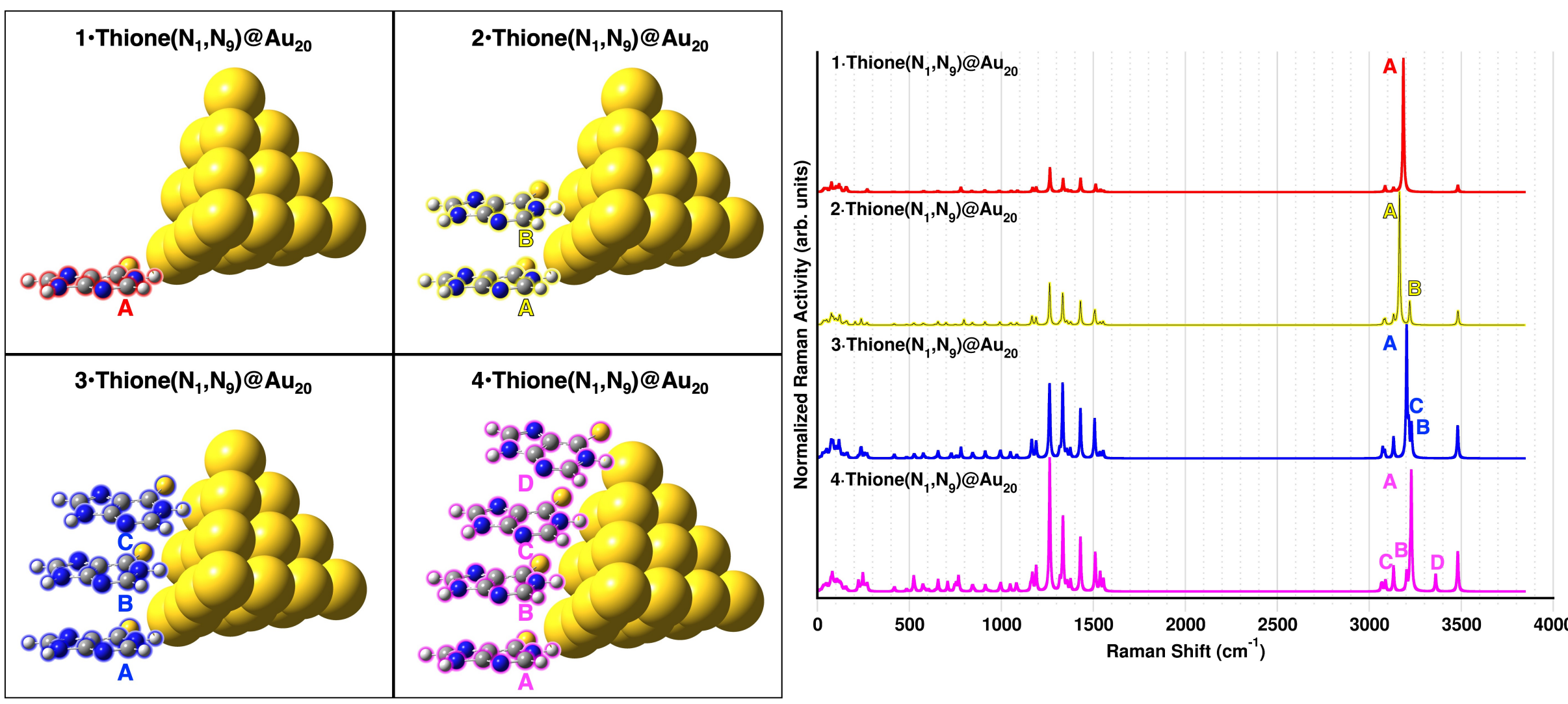

Figure S7. Left: Optimized structures of Thione(N_1,N_9)@Au_{20} complexes containing one to four adsorbed 6-mercaptopurine units computed at the B3LYP/6-311+G(d,p)/GD3BJ/C-PCM level of theory. The binding free energies are reported in Table 5. Right: corresponding normalized Raman spectra of Thione(N_1,N_9)@Au_{20} complexes with increasing number of adsorbed 6-mercaptopurine units.

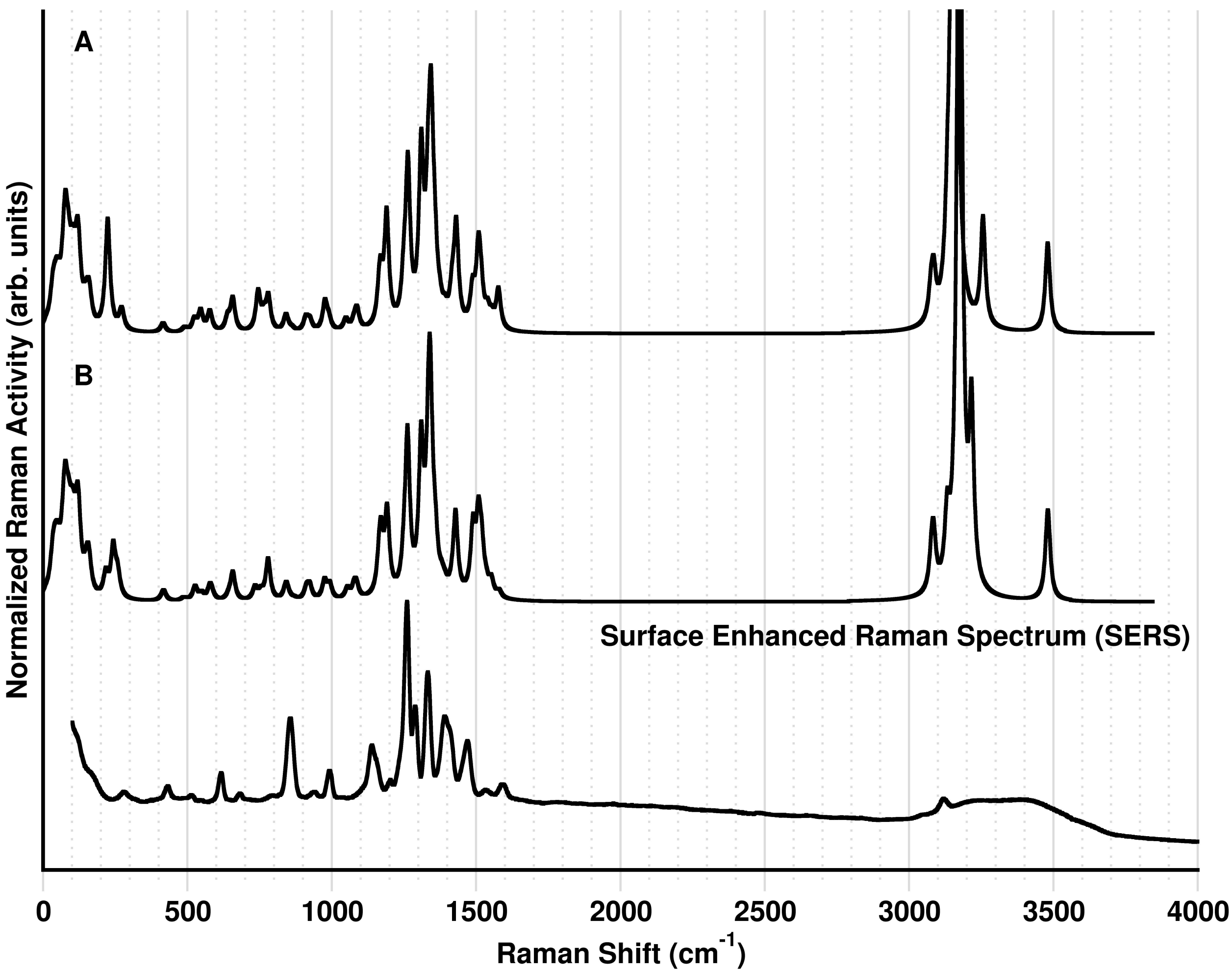

Figure S8. Normalized Raman spectra of three different combinations of Thione(N_1,N_9)/(N_1,N_7)@Au_{20} complexes containing adsorbed 6-mercaptopurine units in a 1:1 ratio (labeled **A**-**B**) computed at the B3LYP/6-311+G(d,p)/GD3BJ/C-PCM level of theory. The experimental SERS spectrum is reported at the bottom for comparison.
A: Thione(N_1,N_9):Thione(N_1,N_7)@Au_{20}
B: Thione(N_1,N_7):Thione(N_1,N_9)@Au_{20}

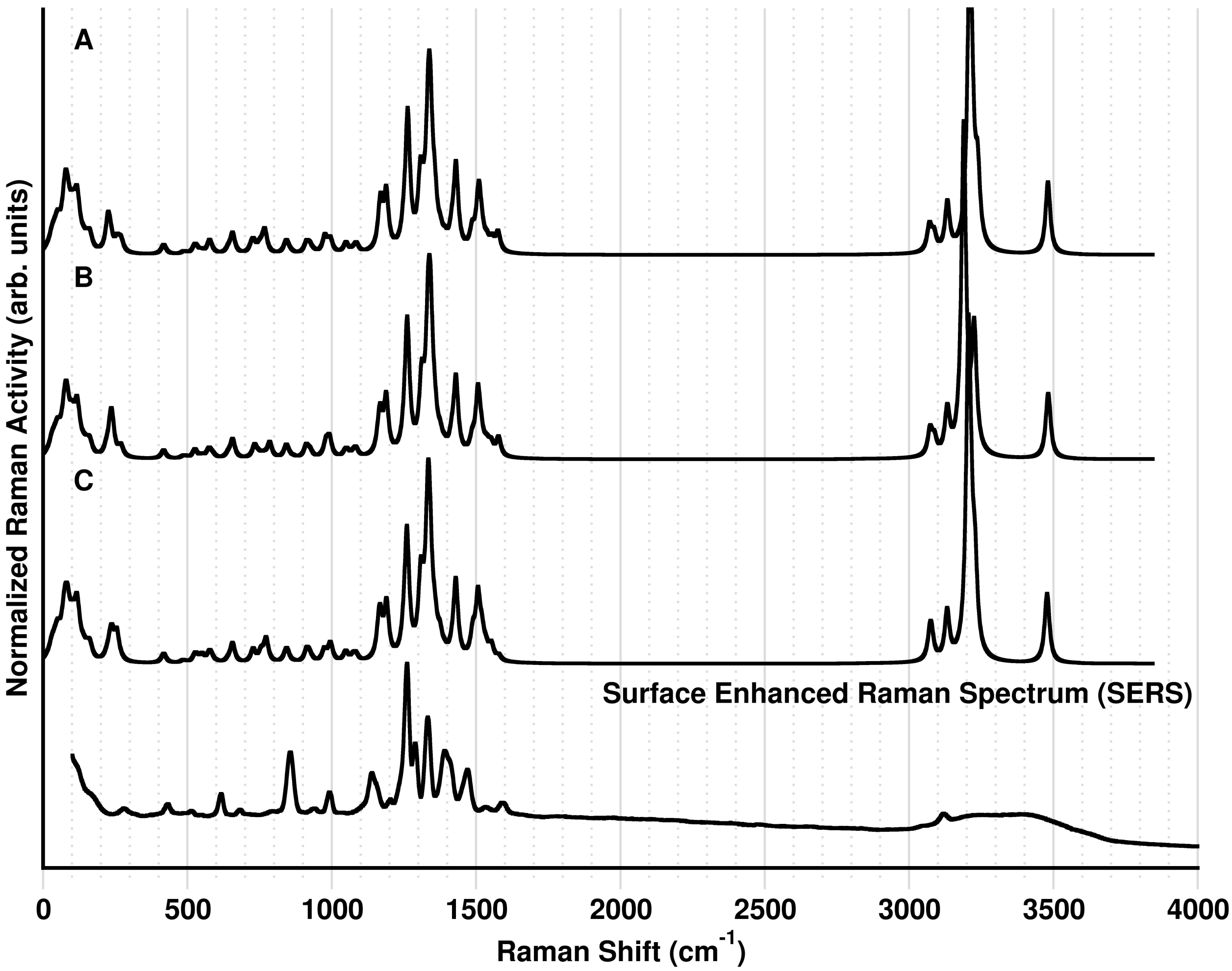

Figure S9. Normalized Raman spectra of three different combinations of Thione(N_1,N_9)/(N_1,N_7)@Au_{20} complexes containing adsorbed 6-mercaptopurine units in a 2:1 ratio (labeled **A**-**C**) computed at the B3LYP/6-311+G(d,p)/GD3BJ/C-PCM level of theory. The experimental SERS spectrum is reported at the bottom for comparison.
A: 2·Thione(N_1,N_9):Thione(N_1,N_7)@Au_{20}
B: Thione(N_1,N_9):Thione(N_1,N_7):Thione(N_1,N_9)@Au_{20}
C: Thione(N_1,N_7):2·Thione(N_1,N_9)@Au_{20}

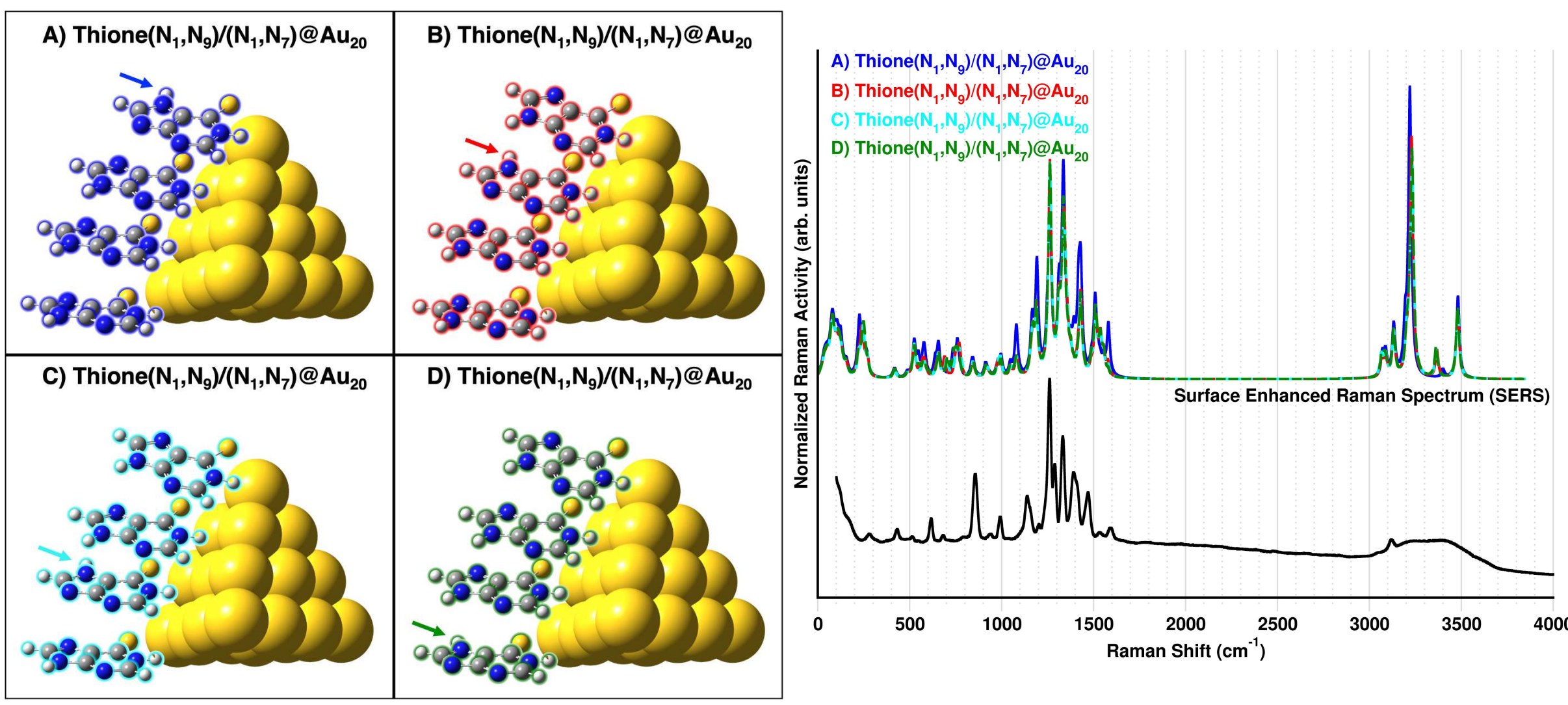

Figure S10. Left: Optimized structures of Thione(N_1,N_9)/(N_1,N_7)@Au_{20} complexes containing adsorbed 6-mercaptopurine units in a 3:1 ratio computed at the B3LYP/6-311+G(d,p)/GD3BJ/C-PCM level of theory. The arrow shows the Thione(N_1,N_7) site. Right: corresponding normalized Raman spectra of the same complexes compared to the experimental SERS spectrum (black trace)).

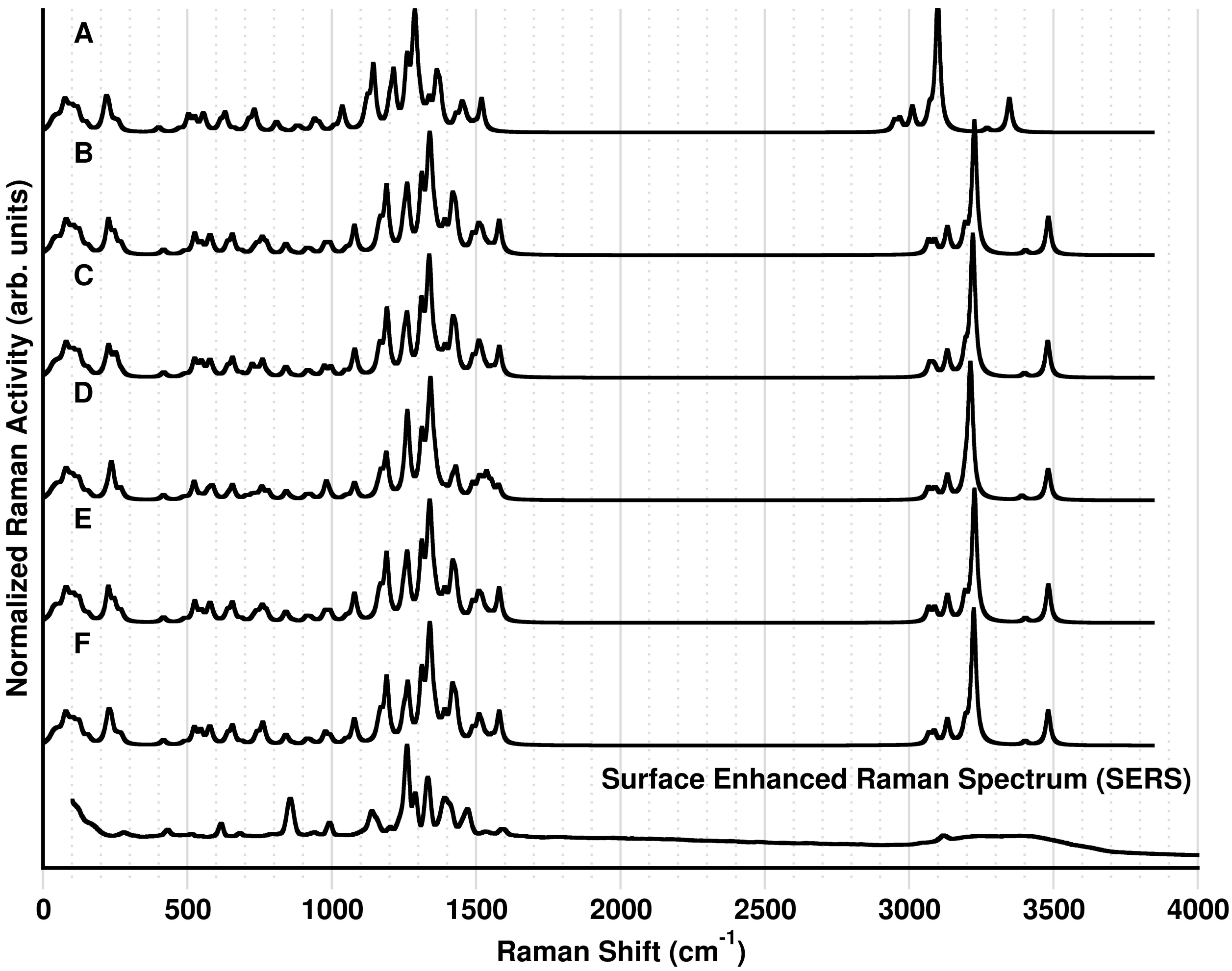

Figure S11. Normalized Raman spectra of six different combinations of Thione(N_1,N_9)/(N_1,N_7)@Au_{20} complexes containing adsorbed 6-mercaptopurine units in a 2:2 ratio (labeled **A**-**F**) computed at the B3LYP/6-311+G(d,p)/GD3BJ/C-PCM level of theory. The experimental SERS spectrum is reported at the bottom for comparison.

A: 2·Thione(N_1,N_9):2·Thione(N_1,N_7)@Au_{20}

B: Thione(N_1,N_9):Thione(N_1,N_7):Thione(N_1,N_9):Thione(N_1,N_7)@Au_{20}

C: Thione(N_1,N_7):2·Thione(N_1,N_9):Thione(N_1,N_7)@Au_{20}

D: Thione(N_1,N_9):2·Thione(N_1,N_7):Thione(N_1,N_9)@Au_{20}

E: 2·Thione(N_1,N_7):2·Thione(N_1,N_9)@Au_{20}

F: Thione(N_1,N_7):Thione(N_1,N_9):Thione(N_1,N_7):Thione(N_1,N_9)@Au_{20}

Table S10. Experimental Raman shifts (in wavenumbers, cm^{-1}) observed in SERS spectrum.

Mode	SERS
1	279
2	432
3	513
4	546
5	617
6	682
7	857
8	940
9	993
10	1140
11	1203
12	1261
13	1289
14	1333
15	1392
16	1410
17	1470
18	1533
19	1593

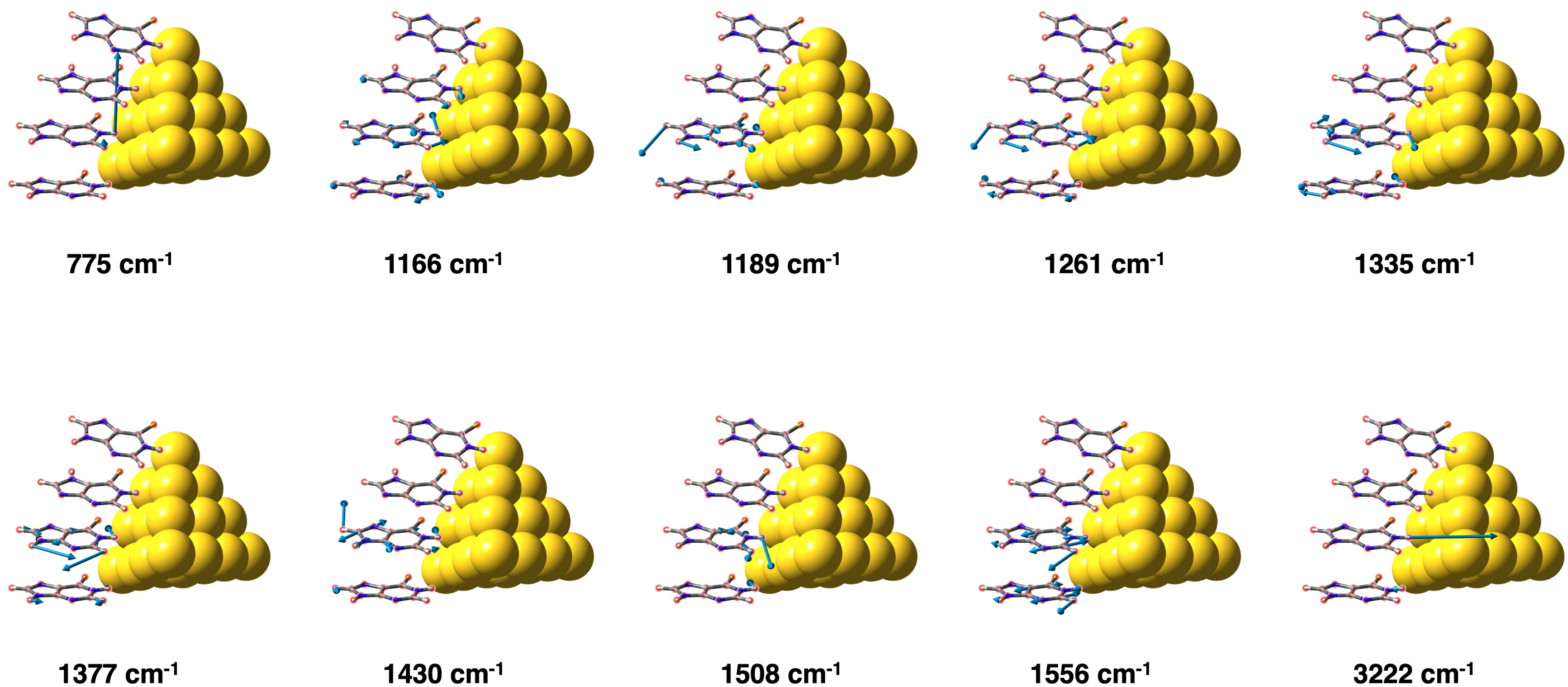

Figure S12. Normal modes displacement vectors computed at B3LYP/6-311+G(d,p)/GD3BJ/C-PCM level of theory associated to the Raman peaks of cluster **B) Thione(N_1,N_9)/(N_1,N_7)@Au_{20}** reported in Figure 9 and Tab. 7.

References

(1) Ivanov, A. Y.; Stepanian, S.; Karachevtsev, V.; Adamovicz, L. Tautomers of 6-thiopurine in low-temperature Ar matrices: FTIR spectroscopy analysis and quantum mechanical calculations. *Low Temperature Physics* **2022**, *48*, 301–310.

(2) Huyen, D. T.; Bui, T. Q.; Si, N. T.; Nhat, P. V.; Quy, P. T.; Nhung, N. T. A. Theoretical study of the binding mechanism between anticancerous drug mercaptopurine and gold nanoparticles using a cluster model. *Journal of Molecular Modeling* **2023**, *29*, 307.

(3) Pan, X.-L.; Cui, F.-C.; Liu, J.-Y. Quantum mechanical/molecular mechanical molecular dynamics and free energy simulations of the Thiopurine S-Methyltransferase reaction with 6-mercaptopurine. *The Journal of Physical Chemistry B* **2011**, *115*, 8033–8037.

(4) Lapiński, L.; Nowak, M. J.; Kwiatkowski, J. S.; Leszczynski, J. Phototautomeric reaction, tautomerism, and infrared spectra of 6-thiopurine. Experimental matrix isolation and quantum-mechanical (conventional ab initio and density-functional theory) studies. *The Journal of Physical Chemistry A* **1999**, *103*, 280–288.
